# Anabaenopeptins from Cyanobacteria in Freshwater Bodies of Greece

**DOI:** 10.3390/toxins14010004

**Published:** 2021-12-21

**Authors:** Sevasti-Kiriaki Zervou, Triantafyllos Kaloudis, Spyros Gkelis, Anastasia Hiskia, Hanna Mazur-Marzec

**Affiliations:** 1 Laboratory of Photo-Catalytic Processes and Environmental Chemistry, Institute of Nanoscience & Nanotechnology, National Centre for Scientific Research “Demokritos”, Patriarchou Grigoriou E & 27 Neapoleos Str., 15310 Athens, Greece; t.kaloudis@inn.demokritos.gr (T.K.); a.hiskia@inn.demokritos.gr (A.H.); 2 Department of Botany, School of Biology, Aristotle University of Thessaloniki, 54124 Thessaloniki, Greece; sgkelis@bio.auth.gr; 3 Division of Marine Biotechnology, University of Gdansk, Al. Marszałka Piłsudskiego 46, 81-378 Gdynia, Poland; hanna.mazur-marzec@ug.edu.pl

**Keywords:** anabaenopeptins, LC–qTRAP MS/MS, fragmentation spectra, structure elucidation, cyanopeptides, cyanobacterial metabolites, Greek freshwaters, cyanobacteria

## Abstract

Cyanobacteria are photosynthetic microorganisms that are able to produce a large number of secondary metabolites. In freshwaters, under favorable conditions, they can rapidly multiply, forming blooms, and can release their toxic/bioactive metabolites in water. Among them, anabaenopeptins (APs) are a less studied class of cyclic bioactive cyanopeptides. The occurrence and structural variety of APs in cyanobacterial blooms and cultured strains from Greek freshwaters were investigated. Cyanobacterial extracts were analyzed with LC–qTRAP MS/MS using information-dependent acquisition in enhanced ion product mode in order to obtain the fragmentation mass spectra of APs. Thirteen APs were detected, and their possible structures were annotated based on the elucidation of fragmentation spectra, including three novel ones. APs were present in the majority of bloom samples (91%) collected from nine Greek lakes during different time periods. A large variety of APs was observed, with up to eight congeners co-occurring in the same sample. AP F (87%), Oscillamide Y (87%) and AP B (65%) were the most frequently detected congeners. Thirty cyanobacterial strain cultures were also analyzed. APs were only detected in one strain (*Microcystis ichtyoblabe*). The results contribute to a better understanding of APs produced by freshwater cyanobacteria and expand the range of structurally characterized APs.

## 1. Introduction

Anabaenopeptins (APs) are cyanobacterial metabolites with a cyclic peptide structure [1]. The presence of APs has been reported in freshwater [2,3,4,5,6] and marine cyanobacterial blooms [7,8,9], as well as in terrestrial environments, including the leaves of plants in a coastal forest [10] and the terrestrial mat in a bamboo forest [11]. APs are produced by freshwater, marine and terrestrial cyanobacteria [12] mainly belonging to the genera *Planktothrix* [13,14], *Anabaena* [15,16,17,18], *Microcystis* [19,20,21,22,23] and *Nostoc* [24,25]. They especially belong to the species *Planktothrix* (*Oscillatoria*) *agardhii* [3,26,27,28,29,30,31,32], *Planktothrix rubescens* [30,31,33,34], *Anabaena* (*Dolichospermum*) *flos-aquae* [35], *Anabaena lemmermannii* [36], *Microcystis aeruginosa* [3,37,38,39], *Microcystis flos-aquae* [36], *Microcystis ichthyoblabe*, *Microcystis wesenbergii* [19] and *Nostoc punctiforme* [40], as well as *Aphanizomenon flos-aquae* [41], *Nodularia spumigena* [40,42,43,44,45], *Woronichinia naegeliana* [46,47] and *Woronichinia compacta* [48]. Additionally, the cyanobacteria *Lyngbya* sp. [49], *Lyngbya confervoides* [50], *Schizothrix* sp. [51], *Tychonema* sp. [52] and *Brasilonema* sp. [11] are known APs producers. APs are also produced by symbiotic cyanobacteria that have been isolated from the marine sponges *Theonella* sp. [53,54,55], *Theonella swinhoei* [56], *Psammocinia aff. bulbosa* [57], and from the lithistid family *Theonellidae* [58].

**Figure 1:**
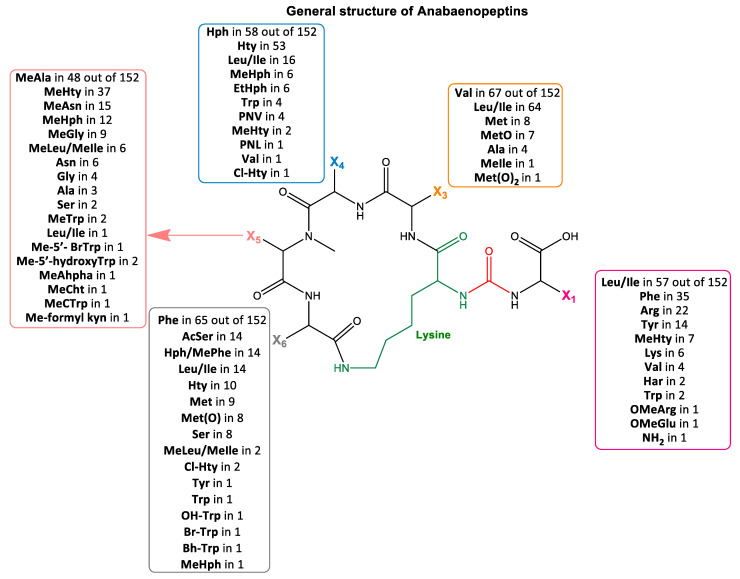
General structure of anabaenopeptins (APs) and an overview of their variable amino acids. Ala = alanine, AcSer = acetyl-serine, Arg = arginine, Asn = asparagine, Bh-Trp = 2-bromo-5-hydroxy-tryptophan, Br-Trp = bromo-tryptophan, Cl-Hty = chloro-homotyrosine, EtHph = ethyl-homophenylalanine, Gly = glycine, Har = homoarginine, Hph = homophenylalanine, Hph/MePhe = homophenylalanine/methyl-phenylalanine (isobaric compounds), Hty = homotyrosine, Leu/Ile = leucine/isoleucine (isobaric compounds), Lys = lysine, Met = methionine, MetO = methionine sulfoxide, Met(O)_2_ = methionine sulfone (S-dioxide), Me-5’-BrTrp = mehtyl-5’-bromo-tryptophan, Me-5’-hydroxyTrp = mehtyl-5’-hydroxy-tryptophan, MeAhpha = N-methyl -2-amino-6-(hydroxyl phenyl) hexanoic acid, MeAla = methyl-alanine, MeAsn = methyl-asparagine, MeCht = 6-chloro-5-hydroxy-N-methyl-tryptophan, MeCTrp = 6-chloro-N-methyl-tryptophan, Me-formyl kyn = methyl-formyl kynurenine, MeGly = methyl-glycine, MeHph = methyl-homophenylalanine, MeHty = methyl-homotyrosine, MeLeu/MeIle = methyl-leucine/methyl-isoleucine (isobaric compounds), MeTrp = methyl-tryptophan, OMeArg = arginine methyl ester, OMeGlu = glutamic acid methyl ester, OHTrp = hydroxyl-tryptophan, Phe = phenylalanine, PNV = 5-phenylnorvaline, PNL = 6-phenylnorleucine, Ser = serine, Trp = tryptophan, Tyr = tyrosine, Val = valine.

APs are hexapeptides with the general structure X_1_-CO-[Lys-X_3_-X_4_-MeX_5_-X_6_], characterized by the presence of the amino acid lysine (Lys), which contributes to ring formation by an *N*-peptide bond with the carboxy group of amino acid X_6_ and a C-peptide bond with the amino group of amino acid X_3_. A side chain of one amino acid unit is attached to a five-peptide ring via an ureido bond between the a-N of Lys and the a-N of the side chain amino acid [1,35] (Figure 1). Apart from Lys, all other amino acids are variable, while the amino acid in position X_5_ is usually *N*-methylated and the amino acid in position 4 is usually in homo form (Table S1).

The first characterized APs (i.e., Anabaenopeptin A and Anabaenopeptin B) were isolated in 1995 by Harada et al. from the freshwater cyanobacterial strain *Anabaena flos-aquae* NRC 525-17 [35], from which APs were named. Structural variants of APs are also known by other names such as Nodulapeptins [42], Oscillamides [27,30], Konbamide [54], Keramamides [53,55], Ferintoic acids [59], Mozamides [58], Schizopeptin [51], Brunsvicamides [52], Psymbamide A [57], Pompanopeptin B [50], Paltolides [56], Lyngbyaureidamides [49] and Nostamides [24,40]. These names were mainly based on the producing taxon or on the geographic location of discovery, complemented with suffixes describing the variety. As a consequence, nomenclature of this class is not fully systematic. Newly identified APs may also contain their molecular mass as part of their name for consistency; however, this approach may be problematic because several variants can have the same molecular mass due to the large number of possible combinations of the variable amino acid residues in the structure (Table S1).

The biosynthesis of APs is performed via nonribosomal peptide synthesis (NRPS) pathways (*aptABCD*) encoded in the genomes of a variety of cyanobacteria [40,60,61,62] and assembled by an NRPS enzyme complex, which has a modular structure [61]. Synthesis is performed stepwise by modules that each contain specific functional domains for the elongation of the peptide sequence through adenylation and thiolation of the activated amino acid residues, ring formation by the epimerization of Lys and *N*-methylation of amino acid X_5_ [61]. The gene clusters of APs can encode either a single starter module, as in *Nostoc* and *Nodularia*, or two alternative loading modules, as in *Anabaena* sp. 90, allowing the simultaneous synthesis of multiple AP variants [40]. Possibly, due to the relaxed substrate specificity of NRPSs, numerous structural variants of cyanobacterial peptides may be generated [61]. Up to now, more than 150 AP congeners have been reported in the literature (Table S1).

Bioactivity studies have shown that APs can inhibit the enzymes responsible for the regulation of several physiological and metabolic processes [63]. Specifically, AP congeners can inhibit carboxypeptidase A [2,9,29,32,41,64], serine proteases (chymotrypsin [27,65], trypsin [51,65] and elastase [65,66,67]), serine/threonine protein phosphatases (PP1 and PP2A [9,30,68]) and *Mycobacterium tuberculosis* enzyme MptpB [52]. Of great pharmacological interest is their high activity against the thrombin activatable fibrinolysis inhibitor (TAFIa) (carboxypeptidase), resulting in the stimulation of fibrin clot degradation, which may help to prevent thrombosis [69]. On the other hand, APs can cause toxic effects on microorganisms such as the nematode *Caenorhabditis elegans* [70], the amoeba *Acanthamoeba castellanii* [36] and the planktic crustacean *Daphnia pulex* [71]. Furthermore, APs could possibly control cyanobacterial population density as the presence of APs (i.e., Anabaenopeptin B and Anabaenopeptin F) has been correlated with the triggering of cell lysis that ends up in the collapse of cyanobacterial blooms [72].

The occurrence of APs in cyanobacterial blooms and cultured strains from freshwater bodies has been reported more frequently during recent years in several countries worldwide, including Japan [2,26,27,28], Germany [3,13,19,20], Finland [16,18], Norway [73,74], Poland [47,75,76], Slovenia [33], Czech Republic [22,77], Austria [34], Hungary [23], Switzerland [78], Spain [4,6,79], Portugal [37,38], Italy [80,81,82,83,84], France [85,86], United Kingdom [85,87], Turkey [44], Israel [21,39,88], Brazil [89], Canada [59,86,90], USA [64,91,92,93,94], New Zealand [95], and India [96]. Recent studies indicate that APs could be more abundant in freshwaters than other toxic cyanobacterial metabolites such as the known cyanotoxins microcystins [6,87,90]. APs have also been detected in wild-caught fishes (fish muscles) from Pike River, Canada [97].

Structural characterization of cyanobacterial metabolites, including APs, is an emerging issue due to the great diversity of molecules, their bioactivities and possible effects on ecosystems and on human health. Nuclear magnetic resonance (NMR), after the isolation of the compound, usually from a cyanobacterial strain culture, has been used for the structural elucidation of APs e.g., [2,26,27,35]. Mass spectrometric (MS) techniques such as Matrix-Assisted Laser Desorption/Ionization Time-of-Flight (MALDI-TOF) [3,22,95] or MS coupled with liquid chromatography such as liquid chromatography–hybrid triple quadrupole/linear ion trap mass spectrometry (LC–qTRAP) [8,23,25,40,45,48] or liquid chromatography–hybrid triple quadrupole/Time-of-Flight (LC–qTOF) [24,44] are nowadays widely used as they can be applied directly to extracts of field samples or strain cultures. A significant indicator of APs′ fragmentation spectrum is the characteristic fragment ion of lysine (Lys) at *m*/*z* 84 [3]. The typical fragmentation pattern of APs includes the loss of the amino acid and the CO of the side chain, resulting in the peptide ring ion [3,10,44].

Information regarding the presence of APs in Greek freshwater bodies is limited. Only three monitoring studies have been conducted so far, targeting no more than three AP congeners [5,98,99]. In the present study, an untargeted analysis approach utilizing a LC–qTRAP method was applied for the investigation of APs′ presence in cyanobacteria from Greece. The main aims were (i) to report, for the first time, the structural diversity of APs in cyanobacterial bloom samples collected from lakes of Greece, (ii) to assess the ability of Greek freshwater cyanobacterial strains to produce APs and (iii) to identify the possible new structures of APs, contributing to a better understanding of the existing variety of these hexapeptide cyanobacterial metabolites.

## 2. Results and Discussion

### 2.1. Structural Elucidation of Anabaenopeptins 

**Figure 2:**
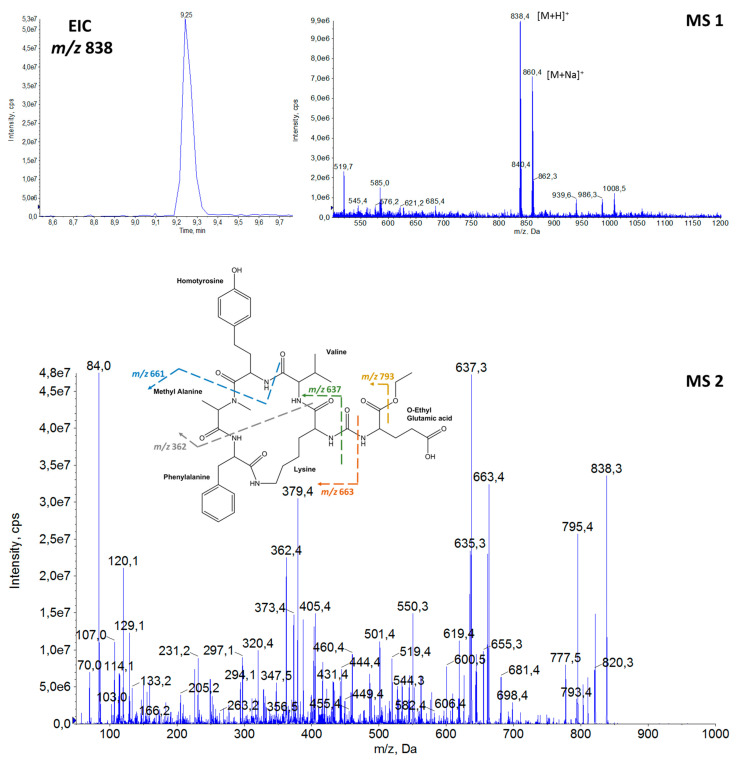
Extracted ion chromatogram (EIC) at *m*/*z* 838, full scan spectrum (MS1) at 9.25 min, fragmentation mass spectrum (MS2) and proposed structure of the new **AP 837** with [M + H]^+^ at *m/z* 838. (*m/z* 820 = [M + H − H_2_O]^+^, *m/z* 793 = [M + H − OCH_2_CH_3_]^+^, *m/z* 663 = [M + H − OEtGlu]^+^, *m/z* 661 = [M + H − Hty]^+^, *m/z* 637 = [Lys-Val-Hty-MeAla-Phe + H]^+^, *m/z* 635 = [M + H − OEtGlu − CO]^+^, *m/z* 562 = [M + H − Val-Hty]^+^, *m/z* 460 = [MeAla-Phe-Lys-Val + H]^+^, *m/z* 405 = [MeAla-Phe-Lys-CO-NH_2_ + H]^+^, *m/z* 362 = [Val-Hty-MeAla + H]^+^, *m/z* 320 = [Phe-Lys-CO-NH_2_ + H]^+^ and/or [Lys-Val-Hty + H]^+^, *m/z* 150 = Hty immonium ions, *m/z* 120 = Phe immonium ion, *m/z* 107 = Hty related ion, *m/z* 102 = Glu immonium ion, *m/z* 84 = Lys immonium ion).

**Figure 3:**
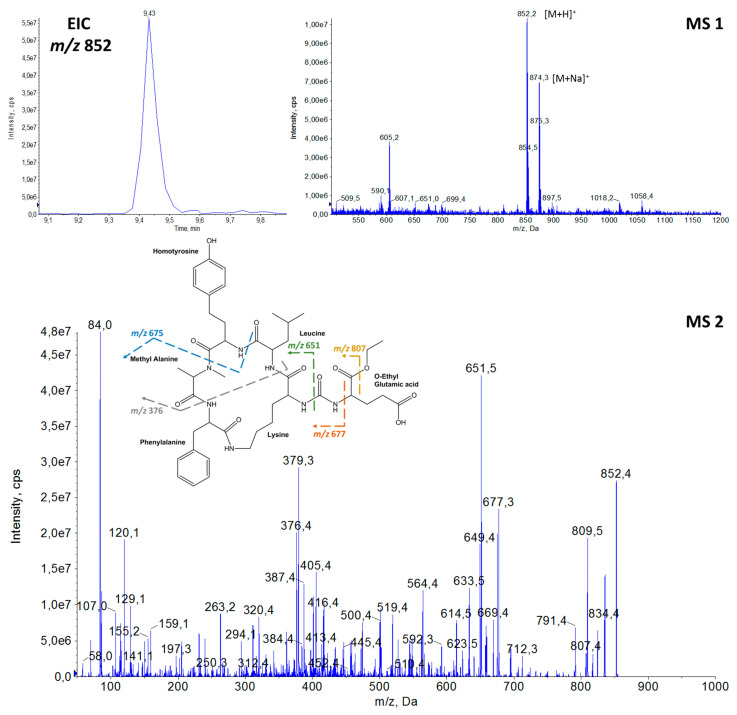
Extracted ion chromatogram (EIC) at *m*/*z* 852, full scan spectrum (MS1) at 9.43 min, fragmentation mass spectrum (MS2) and proposed structure of the new **AP 851** with [M + H]^+^ at *m/z* 852. (*m/z* 834 = [M + H − H_2_O]^+^, *m/z* 807 = [M + H − OCH_2_CH_3_]^+^, *m/z* 677 = [M + H − OEtGlu]^+^, *m/z* 675 = [M + H − Hty]^+^, *m/z* 651 = [Lys-Leu/Ile-Hty-MeAla-Phe + H]^+^, *m/z* 649 = [M + H − OetGlu − CO]^+^, *m/z* 564 = [Phe-Lys-Leu/Ile-Hty + H]^+^, *m/z* 405 = [MeAla-Phe-Lys-CO-NH_2_ + H]^+^, *m/z* 376 = [Leu/Ile-Hty-MeAla + H]^+^, *m/z* 320 = [Phe-Lys-CO-NH_2_ + H]^+^, *m/z* 263 = [MeAla-Hty + H]^+^, *m/z* 150 = Hty immonium ions, *m/z* 120 = Phe immonium ion, *m/z* 107 = Hty related ion, *m/z* 102 = Glu immonium ion, *m/z* 86 = Leu/Ile immonium ion, *m/z* 84 = Lys immonium ion).

**Figure 4:**
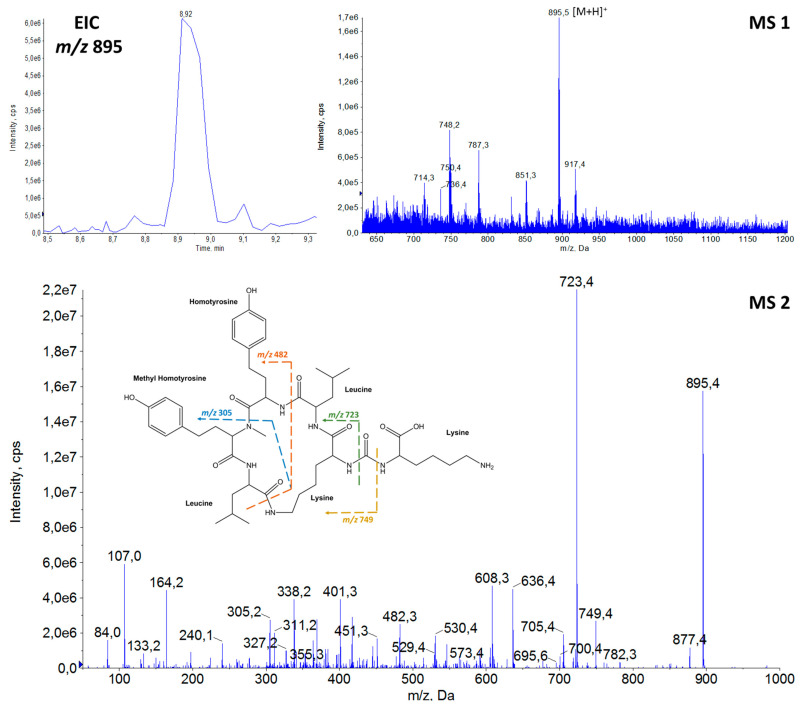
Extracted ion chromatogram (EIC) at *m*/*z* 895, full scan spectrum (MS1) at 8.92 min, fragmentation mass spectrum (MS2) and proposed structure of the new **AP 894** with [M + H]^+^ at *m/z* 895. (*m/z* 749 = [M + H − Lys]^+^, *m/z* 723 = [M + H − Lys − CO]^+^, *m/z* 705 = [Lys-Leu/Ile-Hty-MeHty-Lue/Ile − H_2_O + H]^+^ or [M + H − Lys − CO − H_2_O]^+^, *m/z* 636 = [CO-Lys-Leu/Ile-Hty-MeHty]^+^, *m/z* 608 = [Lys-Leu/Ile-Hty-MeHty]^+^, *m/z* 530 = [Leu/Ile-Lys-Leu/Ile-Hty]^+^, *m/z* 482 = [Leu/Ile-Hty-MeHty + H]^+^, *m/z* 369 = [Hty-MeHty + H]^+^, *m/z* 305 = [MeHty-Leu/Ile + H]^+^, *m/z* 240 = [Lys-Leu/Ile + H]^+^, *m/z* 164 = MeHty immonium ion, *m/z* 107 = Hty related ion, *m/z* 84 = Lys immonium ion).

Thirteen APs were detected in the samples of cyanobacteria from Greek freshwater bodies. The elucidation of proposed AP structures was based on their precursor ions from full scan (MS1) (Table S1) and fragmentation (MS2) spectra, enabling annotation of the compounds [100]. Among them, the possible structures of three AP congeners were proposed for the first time in the frame of the present study. The amino acid sequences of the detected APs with their precursor ions [M + H]^+^ and the retention time (tR) are provided in Table 1. The proposed structures, extracted ion chromatograms (EIC), full scan spectra (MS1) and fragmentation mass spectra (MS2), of the three newly annotated APs are shown in Figure 2, Figure 3 and Figure 4, while the elucidation of their spectra are provided in the relevant captions.

The detection of APs was based on the diagnostic fragment ion of lysine (Lys) *m*/*z* 84 [3], which was present in the fragmentation spectra of all APs (Figure 2, Figure 3 and Figure 4 and Figures S2–S11). Structural elucidation of APs was based on fragmentation patterns described in previous studies [3,9,10,23,25,44,48] and on immonium ions of the common amino acids. In Table 1, both leucine (Leu) and isoleucine (Ile) are provided in the proposed AP sequences as these amino acids are isobaric compounds with the same chemical formula (C_6_H_13_NO_2_) and they could not be distinguished.

Generally, one of the intense ions that is always present in the fragmentation spectrum of APs is the ion formed by the loss of the side chain amino acid, i.e., [M + H − X_1_]^+^. Fragment ions [M + H − X_3_]^+^ and [M + H − X_4_]^+^ are also commonly found in the APs′ spectra. Furthermore, among the most intense fragment ions of APs is the five-peptide ring ion generated after the loss of the side chain, i.e., [Lys-X_3_-X_4_-MeX_5_-X_6_ + H]^+^.

The characteristic ions of annotated APs during this study were *m*/*z* 635 = [Lys-Leu/Ile-Hph-MeAla-Phe + H]^+^ for AP 842 and AP 870; *m*/*z* 637 = [Lys-Val-Hty-MeAla-Phe + H]^+^ for AP B, AP 837, AP A and AP 872; and *m*/*z* 651 = [Lys-Leu/Ile-Hty-MeAla-Phe + H]^+^ that was present in the fragmentation spectra of AP F, AP 851, Osc Y and AP 886. Other common fragment ions of elucidated AP structures were *m*/*z* 320 = [Phe-Lys-CO-NH_2_ + H]^+^ and *m*/*z* 405 = [MeAla-Phe-Lys-CO-NH_2_ + H]^+^ (Figure 2, Figure 3, Figures S4, S5, S7–S10) that suggested the presence of Phe amino acid in position X_6_ and MeAla amino acid in position X_5_.

The fragment ions *m*/*z* 550 = [MeAla-Phe-Lys-CO-Tyr − H_2_O]^+^ and *m*/*z* 568 = [MeAla-Phe-Lys-CO-Tyr]^+^ were characteristic of APs with Tyr as the side chain, i.e., AP 842, AP A and Osc Y (Figures S4, S5 and S7), while the fragment ions *m*/*z* 578 = [MeAla-Phe-Lys-CO-MeHty − H_2_O]^+^ and *m*/*z* 596 = [MeAla-Phe-Lys-CO-MeHty]^+^ indicated the presence of MeHty as the side chain, i.e., AP 870, AP 872 and AP 886 (Figures S8–S10).

Congeners of APs with arginine (Arg) as the side chain amino acid (i.e., AP B, AP F, AP 820, AP KB906) had *m*/*z* 201 = [Arg + CO + H]^+^ as the most intense fragment ion. The other intense fragment ions of the spectra were the [M + H-Arg-CO]^+^ and [M + H-Arg-CO-H_2_O]^+^. The characteristic Lys immonium ion, *m*/*z* 84, was present in the fragmentation spectra with low intensity (Figures S2, S3, S6 and S11).

Immonium ions of amino acids were also significant indicators of the peptide sequences. The presence of Phe was indicated by an intense peak at *m*/*z* 120 and Hph by *m*/*z* 134. Low intensity peaks at *m*/*z* 107 and *m*/*z* 150 suggested the presence of Hty and *m*/*z* 164 of MeHty, while *m*/*z* 136 was attributed to Tyr, *m*/*z* 58 to MeAla and *m*/*z* 86 corresponded to Leu/Ile.

### 2.2. Anabaenopeptins in Cyanobacterial Blooms from Greek Lakes

Samples were collected from nine different lakes of Greece during cyanobacterial bloom events, which were mainly dominated by *Microcystis* and *Dolichospermum* species, and were analyzed for the presence of APs. The detected AP congeners and the dominant cyanobacterial species of each sample are presented in Figure S1, and details are provided in Table 2. In total, thirteen different AP congeners were detected, and their amino acid sequences are shown in Table 1.

The presence of APs was confirmed in the majority of the examined samples (91%). In addition, a large within-sample structural diversity of APs was observed as at least six AP congeners were detected in each of the 11 samples (48% of total samples). Two samples contained only one AP congener. The largest diversity of APs was observed in three samples collected from lakes Kastoria (5 October 1995), Kerkini (3 August 1999) and Zazari (5 August 1999); eight APs were detected in each of them. These samples were dominated by *Microcystis* species (Table 2). A large diversity of APs was also observed in samples collected from lakes Pamvotida, Mikri Prespa, and Vistonida.

APs were not detected in two samples collected from lakes Marathonas and Karla, although cyanobacterial species that possibly produce APs were present in both lakes (i.e., *Microcystis flos-aquae* at Lake Marathonas and *Planktothrix* cf. *agardhii* at Lake Karla).

The most frequently detected APs in Greek freshwater samples were AP F (87% of samples) and Osc Y (87%), followed by AP B (65%) and AP 886 (57%). AP A and AP 872 were also common congeners among the samples. AP 820 and AP KB906 were detected in one sample from Lake Kastoria and Lake Zazari, respectively.

AP 894, whose structure is proposed for the first time in the present study, was detected in two samples collected from lakes Kerkini and Zazari. The newly proposed APs, 837 and 851, were detected in one sample collected from Lake Mikri Prespa (4 November 2014).

In two previous monitoring studies targeting AP A and AP B by HPLC–PDA, in which cyanobacterial bloom samples were collected from up to 36 freshwater bodies of Greece, the presence of APs in lakes Zazari (AP A), Kastoria (AP A and AP B) and Pamvotis (AP A and AP B) was reported [5,98]. In the current study, both AP A and AP B were detected by mass spectrometry in lakes Kastoria, Pamvotis, Zazari, Kerkini, Mikri Prespa and Vistonida, along with several other APs congeners.

According to a three-year monitoring study of the Greek Lake Vegoritis targeting 25 cyanobacterial toxins and peptides, AP B and AP F were found to be the most frequently detected cyanobacterial metabolites; they were present in almost all the samples, followed by Osc Y [99]. These results are in agreement with the current study as AP F, Osc Y and AP B were the most commonly occurring AP congeners in the freshwaters of Greece.

The occurrence of cyanobacterial metabolites, including APs in freshwater blooms, has been investigated in a number of past studies. Analysis by MALDI-TOF MS showed the presence of AP B and AP F in samples collected from lakes in Italy [80,81,102], Germany [3], Spain [79] and Brazil [89]. In samples collected from a waterbody of Poland and analyzed by LC–qTRAP MS/MS, the most abundant AP congener was AP B, followed by AP A, AP F, AP G, Osc Y, AP D and AP 915 [75]. The presence of AP A, AP B, AP F and Osc Y was also confirmed by LC–HRMS in samples collected from the freshwaters of Spain [6] and the Czech Republic [77], while AP B, AP A and Osc Y were identified in samples from the United Kingdom [87]. Based on the results of this study and of previous reports, it appears that AP B and AP F followed by AP A and Osc Y are the most frequently reported APs not only in Greece but also in the European continent.

AP F, Osc Y, AP B and AP A are protease inhibitors that possess activity against carboxypeptidase A and protein phosphatase 1 (PP1) [9,30,64]. AP B and AP F are also highly selective TAFIa inhibitors [69] and elastase inhibitors, with no activity towards chymotrypsin and trypsin [66], while Osc Y have presented inhibitory activity against chymotrypsin [27]. Additionally, AP A, AP B and AP F have had toxicity effects in the nematode *Caenorhabditis elegans* [70]. Even though APs′ toxicity effects on animal models and microorganisms have been reported, there remains a lack of data regarding their toxicity and impact on human health [12].

**Figure 5:**
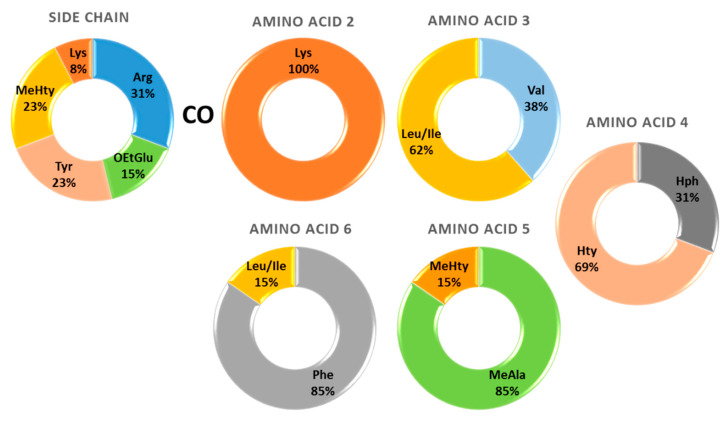
Diversity and frequency of variable amino acids in the structures of anabaenopeptins detected in Greek freshwaters.

APs are the 3rd class of cyanopeptides with the highest structural diversity after microcystins and cyanopeptolins [103]. In the present investigation, thirteen structures of APs from the cyanobacteria of Greek freshwaters were detected, and they had a rather low diversity of variable amino acids (Figure 5). In particular, all the moieties that composed the ring structures were represented by only two different amino acids per site. Even though the diversity was limited, it is interesting that the two amino acids that were determined in each position are among the most commonly found in known AP congeners (Figure 1).

Specifically, the currently known 42 APs from freshwater environments mainly consist of Val (45%) and Ile (29%) in position X_3_, Hty (64%) and Hph (29%) in position X_4_, MeAla (50%) and MeHty (38%) in position X_5_ and Phe (45%) and Ile (24%) in position X_6_ [12]. The 13 APs identified in Greek freshwaters consist of Val (38%) and Ile (62%) in position X_3_, Hty (69%) and Hph (31%) in position X_4_, MeAla (85%) and MeHty (15%) in position X_5_ and Phe (85%) and Ile (15%) in position X_6_ (Figure 5). A comparison of findings strongly supports that the variable amino acids of AP rings determined during this study are consistent with the most common ones of the known APs from freshwaters.

A higher diversity of amino acids was observed in the side chain. Arg (31%) was the most frequent, followed by Tyr (23%), MeHty (23%), OEtGlu (15%) and Lys (8%). Arg and Tyr are present in the side chains of commonly found AP congeners worldwide (i.e., AP B and AP F have Arg; AP A and Osc Y have Tyr). Contrarily, the presence of MeHty as a side chain has been reported for only seven AP congeners that were detected in cyanobacteria from Lake Balaton, Hungary [23]. The proposed side chain of the three novel APs consists of infrequent amino acids (i.e., Lys and OEtGlu). Lys (AP 894) has been determined in the side chain of six known congeners (Figure 1, Table S1), while OEtGlu (AP 837 and AP 851) is proposed for the first time. A previous study reported the presence of OMeGlu occupying the side chain amino acid position in the AP MM823 [65]. In fact, AP MM823 and the newly proposed AP 837 also share the same five-peptide ring structure. Although methylated amino acids are frequently occurring in AP structures, ethylated ones have also been reported [10,25], indicating the metabolomic potential of cyanobacteria.

### 2.3. Anabaenopeptins in Cyanobacterial Strains Isolated from Greek Freshwaters

Thirty cyanobacterial strains from the TAU-MAC culture collection [104], isolated from the freshwaters of Greece, were analyzed in order to evaluate their ability to produce APs (Table S2), i.e., fourteen strains of *Microcystis*, five of *Nostoc*, three of *Jaaginema*, two of *Synechococcus*, and one from the species of the genera *Anabaena*, *Calothrix*, *Chlorogloeopsis*, *Desmonostoc*, *Limnothrix* and *Nodosilinea*. APs were only detected in one strain extract out of the thirty examined. In particular, AP A and Osc Y were identified in the extract of *Microcystis ichtyoblabe* TAU-MAC 0510.

Although AP F and AP B along with Osc Y were the most frequently detected APs in cyanobacterial bloom samples in this study, they were not detected in any of the examined cyanobacterial strains. The diversity of APs in the isolated strains was limited compared to that of bloom extracts. This finding is in agreement with the results of previous studies as it was reported that *Microcystis* strains have a less diverse peptide pattern compared to that of the entire population of a bloom sample from a German lake [19], and that the *Planktothrix agardhii* samples from a Polish freshwater reservoir contained up to seven APs while the two strains isolated from the reservoir contained only one AP [75]. This was rather expected because the diversity of APs in field bloom samples reflects the high diversity of the chemotypes present in water bodies, therefore it cannot be compared with the diversity of the compounds in isolated strains [19,75]. The results of previous chemo-diversity studies of freshwater cyanobacterial strains also indicate the limited presence of AP congeners in the samples. Welker et al. reported the presence of APs in only 9% of 850 examined *Microcystis* colonies with five AP structural variants in total [22] while, in another study, 165 *Microcystis* colonies were examined and only up to four APs were detected in 21% of analyzed samples [20]. Martins et al. have also reported a limited presence of APs in *Microcystis aeruginosa* strains where one to three APs were detected in the 30% of examined strains [38]. Furthermore, in an investigation of 18 *Planktothrix* clonal strains, APs were present in 11 of them, with one, two and three APs present in seven, three and one strain, respectively [13]. The limited presence of APs in cyanobacterial strains may also be correlated with the evidence that cyanobacterial strains could lose the ability to produce cyanopeptides under laboratory conditions [105].

In a previous chemo-diversity study including 24 *Microcystis* strains isolated from the same freshwater blooms or from different populations in various geographical areas (i.e., Netherlands, Scotland, France, Senegal, Burkina Faso), it was found that AP A, AP B, AP F and Osc Y were the most commonly detected AP congeners and were mainly produced by *Microcystis aeruginosa* strains, while all the examined *Microcystis wesenbergii*/*M. viridis* strains did not produce APs. A comparison of the specific chemical footprints of the examined strains showed that the metabolite content was influenced globally by microcystin production rather than sampling locality origins [106]. In another study, it was concluded that AP B and AP E/F were among the principal cyanopeptides detected in 165 *Microcystis* sp. colonies isolated from German lakes and that APs were mostly produced by *Microcystis ichthyoblabe* colonies than by *Microcystis aeruginosa* [20]. According to Fastner et al., AP B, AP F and Osc.Y were the most prominent APs in *Microcystis ichthyoblabe* colonies isolated from a German lake, followed by AP I and AP A, while APs were rarely detected in the *Microcystis aeruginosa* colonies and not detected at all in *Microcystis wesenbergii* colonies [19]. A common conclusion of the above studies was that *Microcystis aeruginosa* colonies predominately produced microcystins; this was in contrast to *Microcystis ichthyoblabe* colonies that mainly produced APs rather than microcystins [19,20]. This is in agreement with the results of the present study where one strain belonging to cyanobacterial species *Microcystis ichthyoblabe* was found to be positive to APs while strains belonging to *Microcystis aeruginosa* and *Microcystis viridis* were negative to APs (Table S2).

In general, AP A, AP B, AP F and Osc Y are the most commonly detected APs both in *Microcystis* and *Planktothrix* strains isolated from several water bodies of European countries, such as Austria [34], the Czech Republic [22], Finland [14], Germany [13,19,20], Norway [74], Portugal [37] and Switzerland [31]. The current study constitutes the first investigation into APs′ presence in several cyanobacterial strains isolated from Greek freshwaters.

## 3. Conclusions

The structural diversity of APs from bloom samples and cultured cyanobacteria strains of Greek freshwaters was investigated for the first time, utilizing LC–qTPAR MS/MS in IDA and EIP modes in order to structurally elucidate APs from their fragmentation spectra. Overall, thirteen APs were annotated, with three of these being reported for the first time (AP 837, AP 851 and AP 894). A variety of APs were found to occur in 21 out of 23 samples from cyanobacterial blooms from seven out of nine lakes that were mainly dominated by *Microcystis* and *Dolichospermum* species. The most frequently occurring APs in bloom samples were AP F and Osc Y, followed by AP B, AP 886 and AP A. On the other hand, in thirty samples of cultured cyanobacterial strains isolated from the freshwater bodies of Greece, APs (AP A and Osc Y) were only found in *Microcystis ichtyoblabe* TAU-MAC 0510. The results of this study are in general agreement with previous studies on the occurrence of APs in European freshwater bodies and contribute to the expansion of the range of known AP congeners by introducing three new AP structures and their mass fragmentation spectra. Considering that APs are a class of cyanobacterial bioactive metabolites that naturally occur in water bodies in high frequency and possibly in significant amounts, the results of this study highlight the need for further assessment of their environmental effects and impacts.

## 4. Materials and Methods

### 4.1. Cyanobacterial Bloom Samples

Samples were collected from nine Greek lakes (Amvrakia, Kastoria, Pamvotida, Kerkini, Zazari, Mikri Prespa, Vistonida, Karla, Marathonas) during episodes of cyanobacterial bloom (Table 2). General characteristics and location of the freshwater bodies are provided in the details of previous studies [98,107,108]. Water samples (100–1500 mL) were collected in airtight polyethylene bottles from the surface layer (0–35 cm) at the margins of the lakes where accumulation of cyanobacteria had been observed from May to October in 1995, 1999, 2000, 2010, 2014 and 2015, as previously described [5,98]. Samples were filtered through Whatman GF/C filters (Millipore, Cork, Ireland), lyophilized and stored at −20 °C until analysis. The cyanobacterial biomass of the samples ranged from 10–1000 mg/L. Dominant cyanobacterial species were characterized by microscopic analysis, as previously reported [5,98,109].

### 4.2. Source and Culture Conditions of Cyanobacterial Strains

Thirty cyanobacterial strains isolated from Greek freshwaters from 1999 to 2010 [109] were identified and provided by Thessaloniki Aristotle University Microalgae and Cyanobacteria (TAU-MAC) Culture Collection [104]. Strains were planktic or benthic; details of their origin and isolation are provided in [109]. Cyanobacterial strains belonging to *Chroococcales, Synechococcales* and *Nostocales* based on polyphasic taxonomy were classified into 10 genera (*Anabaena*, *Microcystis*, *Nostoc*, *Synechococcus*, *Limnothrix*, *Calothrix*, *Nodosilinea*, *Desmonostoc*, *Chlorogloeopsis* and *Jaaginema*) and 16 taxa, as listed in Table S2 [110]. Cyanobacterial strain cultures were grown in BG11 medium with or without nitrogen (BG11_0_ for the nitrogen-fixing strains, see Table S2), shaken manually once per day and maintained at 25 °C (*Microcystis* strains) or 20 ± 1 °C (strains of the rest genera) under cool white light fluorescent lamps (Sylvania Standard F36W/154-T8, SLI, Sylvania, Erlangen, Germany) with a light intensity of 20–25 μmol m^−2^ s^−1^ in a 16/8 h light/dark cycle. At the end of the exponential phase of culture growth (between days 20 and 35, depending on the strain, see Table S2), the whole liquid culture (250 mL) was centrifuged and the cyanobacterial cells collected, lyophilized and stored at −20 °C until analysis. Chlorophyll-*a* was extracted from 5 mL of wet biomass with 95% (*v/v*) acetone solution and spectrophotometrically quantified, as outlined in APHA (2005) [111]. The chlorophyll-*a* concentration of the strains at the time of the collection (as an estimate of their biomass) ranged from 6.21–6.77 mg/L.

### 4.3. Sample Preparation and LC–MS/MS Analysis

Analysis of two different sample types, i.e., cyanobacterial blooms and cyanobacterial strain cultures, was performed. The same amount of each sample type was extracted and analyzed. Lyophilized biomass (~10 mg) of each sample was extracted with 1.5 mL of 75% methanol:25% water assisted by vortexing and sonication in an ice bath for 15 min. After centrifugation (10,000 rpm, 15 min), the supernatants were collected and further centrifuged (10,000 rpm, 5 min) prior to LC–MS/MS analysis.

Untargeted analysis was carried out with an Agilent 1200, liquid chromatography apparatus (Agilent Technologies, Waldboronn, Germany) coupled with a hybrid triple quadrupole/linear ion trap mass spectrometer (QTRAP5500, Applied Biosystems, Sciex; Concorde, ON, Canada) according to Mazur-Marzec et al., 2013 [44]. Chromatographic separation was achieved with a reversed phase column (Zorbax Eclipse XDB-C18 4.6 × 150 mm, 5 μm Agilent Technologies, Santa Clara, CA, USA) applying gradient elution. Mobile phases consisted of (A) acetonitrile and (B) 5% acetonitrile in MilliQ water, both containing 0.1% formic acid; flow rate was 0.6 mL min^−1^ and injection volume was 5 μL. Ionization was performed with electrospray (ESI) source in positive mode. For MS detection, information-dependent acquisition (IDA) mode and enhanced ion product (EIP) mode were applied. In IDA mode, a full scan from 500 to 1200 Da was acquired for detection of the compounds. EIP mode was triggered when the signal of an ion was above a threshold of 500,000 cps; the ions were fragmented in the collision cell (Q2) and fragmentation spectra were recorded from 50 to 1000 Da with a scan speed of 2000 Da s^−1^ and collision energy (CE) of 60 V with collision energy spread (CES) of 20 V. Analyst QS^®^ 1.5.1 software was used for data acquisition and processing. Obtained fragmentation spectra were examined in order to elucidate the structures of occurring APs.

## Figures and Tables

**Table 1:** List of anabaenopeptins (APs) detected in cyanobacterial blooms and cyanobacterial strains from Greek lakes.

	*m*/*z* [M + H]^+^	tR (min)	Name	Amino Acid Sequence							Ref.
				1 (Side Chain)	Ureido Linkage	2	3	4	5	6	
1	821.2	9.4	AP 820	Agr	CO	Lys	Val	Hph	MeAla	Phe	[22]
2	837.4	7.9	AP B	Arg	CO	Lys	Val	Hty	MeAla	Phe	[35]
3	838.3	9.2	AP 837	EtOGlu	CO	Lys	Val	Hty	MeAla	Phe	This study
4	842.3	10.6	AP 842	Tyr	CO	Lys	Ile	Hph	MeAla	Phe	[23]
5	844.2	9.9	AP A	Tyr	CO	Lys	Val	Hty	MeAla	Phe	[35]
6	851.3	8.8	AP F	Arg	CO	Lys	Ile	Hty	MeAla	Phe	[28]
7	852.2	9.4	AP 851	EtOGlu	CO	Lys	Leu/Ile	Hty	MeAla	Phe	This study
8	858.4	10.0	Osc Y	Tyr	CO	Lys	Ile	Hty	MeAla	Phe	[27]
9	870.4	10.8	AP 870	MeHty	CO	Lys	Leu/Ile	Hph	MeAla	Phe	[23]
10	872.3	10.1	AP 872	MeHty	CO	Lys	Val	Hty	MeAla	Phe	[23]
11	886.4	10.2	AP 886	MeHty	CO	Lys	Leu/Ile	Hty	MeAla	Phe	[23]
12	895.6	8.9	AP 894	Lys	CO	Lys	Leu/Ile	Hty	MeHty	Leu/Ile	This study
13	907.3	9.5	AP KB906	Arg	CO	Lys	Ile	Hph	MeHty	Ile	[101]

**Table 2:** Dominant cyanobacterial species and anabaenopeptins detected in samples of cyanobacterial blooms from Greek lakes.

Lake	Sampling Date	Dominant Cyanobacterial Species	Anabaenopeptins Amino Acid Sequences Are Listed in Table 1	Number of Congeners
Amvrakia	10 August 1999	*Microcystis* spp., *Dolichospermum viguieri*	AP F, Osc Y, AP 872, AP 886	4
Amvrakia *	19 August 1999	*Dolichospermum perturbatum*, *Microcystis* spp.	AP F	1
Amvrakia *	19 August 1999	*Dolichospermum perturbatum*	AP F, Osc Y, AP 886	3
Kastoria *	5 October 1995	*Microcystis aeruginosa*, *Microcystis novacekii*, *Microcystis wesenbergii*	AP B, AP 842, AP A, AP F, Osc Y, AP 886	6
Kastoria *	5 October 1995	*Microcystis aeruginosa*, *Microcystis novacekii*, *Microcystis wesenbergii*	AP 820, AP B, AP 842, AP A, AP F, Osc Y, AP 872, AP 886	8
Kastoria	3 July 2000	*Microcystis aeruginosa*, *Microcystis novacekii*	AP B, AP F, Osc Y	3
Kastoria	20 September 2000	*Microcystis aeruginosa*, *Microcystis flos-aquae*	AP B, AP F, Osc Y, AP 872	4
Kastoria	18 September 2014	*Microcystis aeruginosa*, *Microcystis flos-aquae*, *Microcystis* spp., *Pseudanabaena mucicola*	AP B, AP F, Osc Y, AP 886	4
Kastoria	6 October 2015	*Microcystis aeruginosa*, *Microcystis flos-aquae*, *Microcystis novacekii*, *Microcystis ichthyoblabe*	AP B, AP F, Osc Y	3
Kerkini	3 August 1999	*Microcystis* spp., *Microcystis wesenbergii*	AP B, AP 842, AP A, AP F, Osc Y, AP 870, AP 872, AP 886	8
Kerkini	26 August 1999	*Microcystis aeruginosa*, *Dolichospermumspiroides*	AP B, AP A, AP F, Osc Y, AP 872, **AP 894**	6
Mikri Prespa	5 August 1999	*Microcystis* spp., *Microcystis wesenbergii*	AP B, AP F, Osc Y, AP 870, AP 872, AP 886	6
Mikri Prespa	4 November 2014	*Microcystis aeruginosa*	AP B, **AP 837**, AP A, AP F, **AP 851**, Osc Y	6
Pamvotida	22 July 1999	*Microcystis aeruginosa*	AP B, AP 842, AP A, AP F, Osc Y, AP 886	6
Pamvotida	18 August 1999	*Dolichospermum flos-aquae*, *Microcystisaeruginosa*	AP F, Osc Y, AP 886	3
Pamvotida	5 August 2000	*Microcystis aeruginosa*, *Dolichospermum flos-aquae*	Osc Y	1
Pamvotida	17 August 2000	*Dolichospermum flos-aquae*, *Microcystis* spp.	AP F, Osc Y, AP 886	3
Pamvotida *	18 August 2000	*Microcystis* spp., *Dolichospermum flos-aquae*	AP B, AP F, Osc Y, AP 870, AP 872, AP 886	6
Pamvotida *	18 August 2000	*Microcystis* spp., *Dolichospermum flos-aquae*	AP B, AP 842, AP F, Osc Y, AP 872, AP 886	6
Vistonida	2 August 1999	*Microcystis aeruginosa*, *Microcystis* spp.	AP B, AP A, AP F, Osc Y, AP 872, AP 886	6
Zazari	5 August 1999	*Microcystis aeruginosa*, *Microcystis* spp.	AP B, AP 842, AP A, AP F, Osc Y, AP 872, **AP 894**, AP KB906	8
Karla	1 July 2015	*Anabaenopsis elenkinii*, *Raphidiopsis (Cylindrospermopsis) raciborskii*, *Planktothrix* cf. *agardhii*, *Pseudanabaena limnetica*	-	0
Marathonas	26 October 2010	*Microcystis flos-aquae*, *Microcystis viridis*, *Pseudanabaena raphidioides*, *Planktolyngbya limnetica*	-	0

* Samples were collected from two different sampling points for Lake Amrakia (19 August 1999), Lake Kastoria (5 October 1995) and Lake Pamvotida (18 August 2000). **BOLD:** New AP structures proposed in the frame of the present study.

## Data Availability

Data are contained within the article or Supplementary Material.

## Supplementary Materials

The following are available online at https://www.mdpi.com/article/10.3390/toxins14010004/s1, Table S1: List of anabaenopeptins reported in the literature and their amino acid sequence, Table S2: List of the cyanobacterial strains from Greek freshwaters, examined for their ability to produce APs, Figure S1: Anabaenopeptins’ presence in cyanobacterial blooms of Greek freshwaters and the dominant species, Figure S2: Fragmentation mass spectrum of Anabaenopeptin 820 with precursor ion at *m*/*z* 821 [M + H]^+^, Figure S3: Fragmentation mass spectrum of Anabaenopeptin B with precursor ion at *m*/*z* 837 [M + H]^+^, Figure S4: Fragmentation mass spectrum of Anabaenopeptin 842 with precursor ion at *m*/*z* 842 [M + H]^+^, Figure S5: Fragmentation mass spectrum of Anabaenopeptin A with precursor ion at *m*/*z* 844 [M + H]^+^, Figure S6: Fragmentation mass spectrum of Anabaenopeptin F with precursor ion at *m*/*z* 851 [M + H]^+^, Figure S7: Fragmentation mass spectrum of Oscillamide Y with precursor ion at *m*/*z* 858 [M + H]^+^, Figure S8: Fragmentation mass spectrum of Anabaenopeptin 870 with precursor ion at *m*/*z* 870 [M + H]^+^, Figure S9: Fragmentation mass spectrum of Anabaenopeptin 872 with precursor ion at *m*/*z* 872 [M + H]^+^, Figure S10: Fragmentation mass spectrum of Anabaenopeptin 886 with precursor ion at *m*/*z* 886 [M + H]^+^, Figure S11: Fragmentation mass spectrum of Anabaenopeptin KB 906 with precursor ion at *m*/*z* 907 [M + H]^+^.
