# Supplementary material for: Anabaenopeptins from Cyanobacteria in Freshwater Bodies of Greece
Source: Toxins (Basel). 2021 Dec 21;14(1):4. doi: 10.3390/toxins14010004 (PMC8781842; doi:10.3390/toxins14010004)
Supplement: Supplementary file 1 [file toxins-14-00004-s001.zip › toxins-1439109-supplementary.pdf]

Supplementary Materials: Anabaenopeptins from cyanobacteria in freshwater bodies of Greece

Sevasti-Kiriaki Zervou, Triantafyllos Kaloudis, Spyros Gkelis, Anastasia Hiskia and Hanna Mazur-Marzec

Table S1. List of Anabaenopeptins reported in the literature and their amino acid sequence.

	Name	*m/z* [M+H]+	Amino acids sequence							Ref.
			1 (Side chain)	Ureido linkage	2	3	4	5	6	
1	Anabaenopeptin 679	678.3618 [M-H]-	NH_2	CO	Lys	Val	Hty	MeAla	Phe	[1]
2	Anabaenopeptin 731	732.3922	Phe	CO	Lys	Val	Leu	MeGly	AcSer	[2]
3	Anabaenopeptin I	760.4618	Ile	CO	Lys	Val	Hty	MeAla	Leu	[3]
4	Nostamide C	778.4497	Ile	CO	Lys	Val	Hph	MeAla	Phe	[4]
5	Nostamide D	778.4498	Val	CO	Lys	Ile	Hph	MeAla	Phe	[4]
6	Nostamide E	778.4498	Ile	CO	Lys	Ile	Hph	Ala	Phe	[4]
7	Anabaenopeptin 788	789.4425	Val	CO	Lys	Val	Trp	MeAla	Phe	[5]
8	Schizopeptin 791	792	Ile	CO	Lys	Ile	Hph	MeAla	Phe	[6]
9	Anabaenopeptin J	794.4462	Ile	CO	Lys	Val	Hty	MeAla	Phe	[3]
10	Anabaenopeptin 802A	803.4417	Val	CO	Lys	Ile	Trp	MeAla	Phe	[7]
11	Anabaenopeptin 802B	803	Arg	CO	Lys	Val	Hty	MeAla	Leu/Ile	[8]
12	Anabaenopeptin 802C	803.4417	Val	CO	Lys	Leu	Trp	MeAla	Phe	[5]
13	Anabaenopeptin 803	804.4353	Leu/Ile	CO	Lys	Met	Leu	MeIle	Met(O)	[2]
14	Nostamide B	806.4810	Ile	CO	Lys	Ile	Hph	MeAla	Hph	[4]
15	Nodulapeptin 807	808	Ile	CO	Lys	Val	Hph	MeHph	Ser	[9]
16	Anabaenopeptin 807	808	Ile	CO	Lys	Ile	Hty	MeAla	Phe	[10]
17	Anabaenopeptin 808	808	Ile	CO	Lys	Val	Hty	MeAla	Hph	[11]
18	Anabaenopeptin C	809.4629	Lys	CO	Lys	Val	Hty	MeAla	Phe	[12, 13]
19	Anabaenopeptin 809	810	Tyr	CO	Lys	Val	Hty	MeAla	Leu/Ile	[8]
20	Anabaenopeptin KVJ841	812.4348	Tyr	CO	Lys	Ile	Hph	MeGly	Hph	[14]
21	Paltolide A	812.4783	Arg	CO	Lys	Ala	Leu	Leu	Trp	[15]
22	Anabaenopeptin 813	814	Phe	CO	Lys	Val	Hty	MeGly	Phe	[16]
23	Anabaenopeptin 816	817.4425	Ile/Leu	CO	Lys	Ile/Leu	Trp	MeAla	Phe	[5]
24	Anabaenopeptin 820	821.47	Arg	CO	Lys	Val	Hph	MeAla	Phe	[17]
25	Nodulapeptin 821	822	Ile	CO	Lys	Ile	Hph	MeHph	Ser	[9]
26	Anabaenopeptin SA3	823.4718	Lys	CO	Lys	Ile	HTy	Ala	Phe	[18]
27	Anabaenopeptin 824 / Nodulapeptin 823	824	Ile	CO	Lys	Val	Hph	MeHty	Ser	[11, 16]
28	Anabaenopeptin 827 A	828	Tyr	CO	Lys	Val	Hph	MeAla	Phe	[8]
29	Anabaenopeptin 827 B	828.38	Phe	CO	Lys	Val	Hph	Ala	Hty	[19]
30	Anabaenopeptin KVJ827	828.4282	Tyr	CO	Lys	Val	Hph	MeGly	Hph	[14]
31	Anabaenopeptin D	828.4563	Phe	CO	Lys	Val	Hty	MeAla	Phe	[12, 13]
32	Anabaenopeptin 834	835.31	Ile/Leu	CO	Lys	Val	Hph	MeAsn	Hph	[19]
33	Anabaenopeptin SA6	835.4718	Ile	CO	Lys	Ile	Hph	Asn	Phe	[18]
34	Anabaenopeptin SA5	835.4718	Ile	CO	Lys	Val	PNV	Asn	Phe	[18]
35	Anabaenopeptin B	837.4629	Arg	CO	Lys	Val	Hty	MeAla	Phe	[20]
36	Anabaenopeptin 837	838.3	OEtGlu	CO	Lys	Val	Hty	MeAla	Phe	This study
37	Nodulapeptin 839	840	Ile	CO	Lys	Met	Hph	MeHph	Ser	[9]
38	Nostamide A	842	Phe	CO	Lys	Ile	Hph	MeGly	Hty	[21]
39	Anabaenopeptin 841A	842	Phe	CO	Lys	Ile	Hty	MeAla	Phe	[10]
40	Anabaenopeptin 841B	842.4416	Phe	CO	Lys	Val	Hph	MeAla	Hty	[7]
41	Anabaenopeptin KVJ811	842.4449	Phe	CO	Lys	Val	Hph	MeGly	Hph	[14]

42	Paltolide B	842.4888	Arg	CO	Lys	Ala	Leu	MeLeu	OHTrp	[15]
43	Anabaenopeptin 842	842.7	Tyr	CO	Lys	Ile/Leu	Hph	MeAla	Phe	[22]
44	Anabaenopeptin SA12	844.4245	Phe	CO	Lys	Val	HTy	Gly	HTy	[18]
45	Anabaenopeptin A	844.4263	Tyr	CO	Lys	Val	Hty	MeAla	Phe	[20]
46	Anabaenopeptin MM823	846.4056 [M+Na]+	OMeGlu	CO	Lys	Val	Hty	MeAla	Phe	[23]
47	Anabaenopeptin 848	849.34	Ile/Leu	CO	Lys	Val	MeHph	MeAsn	Hph	[19]
48	Anabaenopeptin 848	849.4872	Leu	CO	Lys	Ile	MeHph	MeAsn	Phe	[7]
49	Anabaenopeptin SA7	849.4875	Ile	CO	Lys	Ile	PNV	Asn	Phe	[18]
50	Nodulapeptin 849	850	Ile	CO	Lys	Val	Hph	MeHph	AcSer	[9]
51	Anabaenopeptin 849	850.4818	Lys	CO	Lys	Ile	Hph	MeAsn	Phe	[7]
52	Anabaenopeptin E	851.4763	Arg	CO	Lys	Val	MeHty	MeAla	Phe	[24]
53	Anabaenopeptin DA850	851.4774	Arg	CO	Lys	*allo*-Ile	Hty	MeAla	Phe	[25]
54	Anabaenopeptin B1	851.4779	HArg	CO	Lys	Val	Hty	MeAla	Phe	[26]
55	Anabaenopeptin MM850	851.4794	OMeArg	CO	Lys	Val	Hty	MeAla	Phe	[23]
56	Anabaenopeptin F	851.481	Arg	CO	Lys	Ile	Hty	MeAla	Phe	[24]
57	Nodulapeptin 851	852	Ile	CO	Lys	Val	Hph	MeHph	Met	[8]
58	Anabaenopeptin 851	852.2	OEtGlu	CO	Lys	Leu/Ile	Hty	MeAla	Phe	This study
59	Anabaenopeptin 852	852.8	MeHty	CO	Lys	Val	Hty	MeAla	MeLeu/ MeIle	[22]
60	Anabaenopeptin SA2	853.4572	Arg	CO	Lys	Val	HTy	Ser	Phe	[18]
61	Anabaenopeptin 855 B	856	Phe	CO	Lys	Val	Val	MeHty	MetO	[16]
62	Nodulapeptin 855 A	856	Ile	CO	Lys	Met	Hph	MeHty	Ser	[10]
63	Nodulapeptin 855 B	856	Ile	CO	Lys	MetO	Hph	MeHph	Ser	[10]
64	Anabaenopeptin 855 A	856.4561	Phe	CO	Lys	Ile	Hph	MeAla	Hty	[7]
65	Anabaenopeptin 856	856.7	MeHty	CO	Lys	Val	Hph	MeAla	Phe	[22]
66	Oscillamide Y	858.4396	Tyr	CO	Lys	Ile	Hty	MeAla	Phe	[27]
67	Anabaenopeptin 857 A	858.4405	Phe	CO	Lys	Val	Hty	MeAla	Hty	[7]
68	Nodulapeptin 857 /Anabaenopeptin 857 B	858	Phe	CO	Lys	Val	Hph	MeHty	Ser	[9, 16]
69	Brunsvicamide B	859.5063	Ile	CO	Lys	Ile	Leu	MeTrp	Phe	[28]
70	Anabaenopeptin SA13	860.4194	Tyr	CO	Lys	Val	HTyr	Ser	Phe	[18]
71	Mozamide A	861.4875	Ile	CO	Lys	Val	Leu	Me-5′-hydroxyTrp	Phe	[29]
72	Anabaenopeptin 862 A	863.26	Ile/Leu	CO	Lys	Val	EtHph	MeAsn	Hph	[19]
73	Anabaenopeptin 862 B	863.5028	Leu	CO	Lys	Ile	EtHph	MeAsn	Phe	[7]
74	Anabaenopeptin SA8	863.5031	Ile	CO	Lys	Ile	PNL	Asn	Phe	[18]
75	Nodulapeptin 863	864	Ile	CO	Lys	Ile	Hph	MeHph	AcSer	[9]
76	Anabaenopeptin NZ825	864.4036 [M+K]+	Phe	CO	Lys	Ile	Hph	MeGly	Hph	[30]
77	Lyngbyaureidamide B	864.4255 [M+Na]+	Phe	CO	Lys	Ile	Hty	MeAla	Phe	[31]
78	Anabaenopeptin NZ841	864.4337 [M+Na]+	Phe	CO	Lys	Ile	Hty	MeGly	Hph	[30]
79	Anabaenopeptin 863	864.4972	Lys	CO	Lys	Ile	MeHph	MeAsn	Phe	[7]
80	Anabaenopeptin SA4	864.4984	Lys	CO	Lys	Ile	PNV	Asn	Phe	[18]
81	Anabaenopeptin 864	865.3	Ile/Leu	CO	Lys	Val	MeHph	MeAsn	Hty	[19]
82	Anabaenopeptin KT864	865.4963	Harg	CO	Lys	Ile	Hty	MeAla	Phe	[32]
83	Nodulapeptin 865	866	Ile	CO	Lys	Val	Hph	MeHty	AcSer	[9]
84	Anabaenopeptin T	866.5076	Ile	CO	Lys	Val	Hty	MeHty	Ile	[33]
85	Anabaenopeptin 866	866.7	MeHty	CO	Lys	Ile/Leu	Hty	MeAla	MeLeu/ MeIle	[22]
86	Ferintoic acid A	867.4417	Trp	CO	Lys	Val	Hty	MeAla	Phe	[34]
87	Brunsvicamide A	867.4764 [M+Na]+	Ile	CO	Lys	Val	Leu	MeTrp	Phe	[28]

88	Anabaenopeptin 868	868	Ile	CO	Lys	MetO	Hty	MeHt	Met	[11]
89	Nodulapeptin 867	868	Ile	CO	Lys	Val	Hph	MeHty	Met	[9]
90	Oscillamide B	869.4324	Arg	CO	Lys	Met	Hty	MeAla	Phe	[35]
91	Nodulapeptin 869	870	Phe	CO	Lys	Val	Leu	MeHty	MetO	[16]
92	Anabaenopeptin 869	870.16	Tyr	CO	Lys	Ile/Leu	Hph	MeAla	MeHph	[19]
93	Anabaenopeptin 870	870.7	MeHty	CO	Lys	Ile/Leu	Hph	MeAla	Phe	[22]
94	[Ser^6] Nodulapeptin B	872	Ile	CO	Lys	MetO	Hph	MeHty	Ser	[21]
95	Anabaenopeptin 871	872.4544	Phe	CO	Lys	Ile	Hty	MeAla	Hty	[7]
96	Anabaenopeptin 872	872.6	MeHty	CO	Lys	Val	Hty	MeAla	Phe	[22]
97	Mozamide B	875.5031	Ile	CO	Lys	Ile	Leu	Me-5′-hydroxy Trp	Phe	[29]
98	Anabaenopeptin SA9	876.4062	Phe	CO	Lys	Ile	ClHTy	Gly	Hph	[18]
99	Anabaenopeptin 876	877.16	Ile/Leu	CO	Lys	Ile/Leu	EtHph	MeAsn	Hph	[19]
100	Konbamide	877.3824	Leu	CO	Lys	Ala	Leu	MeLeu	BhTrp	[36]
101	Anabaenopeptin SA10	878.3855	Phe	CO	Lys	Val	HTy	Gly	ClHTyr	[18]
102	Lyngbyaureidamide A	878.4426 [M+Na]$^+$	Phe	CO	Lys	Ile	Hty	MeAla	Hph	[31]
103	Anabaenopeptin 877A	878.4864	Agr	CO	Lys	Ile	Hph	MeAsn	Phe	[7]
104	Anabaenopeptin 877B	878.5122	Lys	CO	Lys	Ile	EtHph	MeAsn	Phe	[7]
105	Nodulapeptin 879	880	Ile	CO	Lys	Ile	Hph	MeHty	AcSer	[10]
106	Anabaenopeptin NZ857	880.4269 [M+Na]$^+$	Phe	CO	Lys	Ile	Hty	MeGly	Hty	[30]
107	Ferintoic acid B	881.4567	Trp	CO	Lys	allo-Ile	Hty	MeAla	Phe	[34]
108	Nodulapeptin 881 A	882	Ile	CO	Lys	Met	Hph	MeHph	AcSer	[10]
109	Nodulapeptin 881 B	882	Ile	CO	Lys	Ile	Hph	MeHty	Met	[10]
110	Anabaenopeptin 882	883.4695	Phe	CO	Lys	Ile	MeHph	MeAsn	Phe	[7]
111	Nodulapeptin 883 A	884	Ile	CO	Lys	Met	Hph	MeHph	Met	[10]
112	Nodulapeptin 883 B	884	Phe	CO	Lys	Val	Hph	MeHph	AcSer	[10]
113	Nodulapeptin 883 C	884	Ile	CO	Lys	Val	Hph	MeHty	MetO	[9]
114	Anabaenopeptin 884	884	Ile	CO	Lys	Val	Hph	MeHty	AcSer	[11]
115	Nodulapeptin 885	886	Phe	CO	Lys	Val	Hph	MeHph	Met	[9]
116	Anabaenopeptin 886	886.7	MeHty	CO	Lys	Ile/Leu	Hty	MeAla	Phe	[22]
117	Anabaenopeptin SA1	890.4888	Arg	CO	Lys	Ile	PNV	Asn	Phe	[18]
118	Anabaenopeptin SA11	892.4012	Phe	CO	Lys	Ile	HTy	Gly	ClHTyr	[18]
119	Anabaenopeptin 891	892.5023	Arg	CO	Lys	Ile	MeHph	MeAsn	Phe	[7]
120	Anabaenopeptin HU892	893.5284	Arg	CO	Lys	Val	Hph	MeHty	Ile	[37]
121	Anabaenopeptin 894	895.6	Lys	CO	Lys	Leu/Ile	Hty	MeHty	Leu/Ile	This study
122	Anabaenopeptin 896	897.4858	Phe	CO	Lys	Ile	EtHph	MeAsn	Phe	[7]
123	[MeHph^5] Nodulapeptin B	898	Ile	CO	Lys	MetO	Hph	MeHph	AcSer	[21]
124	Nodulapeptin C	898	Ile	CO	Lys	Met	Hph	MeHty	AcSer	[21]
125	Brunsvicamide C	899.4692 [M+Na]$^+$	Ile	CO	Lys	Val	Leu	Me-formyl kyn	Phe	[28]
126	Nodulapeptin 899 / Anabaenopeptin 900	900	Phe	CO	Lys	Val	Hph	MeHty	AcSer	[11, 38]
127	Nodulapeptin 899 B	900	Ile	CO	Lys	Val	Hty	MeHty	MetO	[16]
128	[Met^6] Nodulapeptin C	900	Ile	CO	Lys	Met	Hph	MeHty	Met	[21]
129	Anabaenopeptin 899	900.6	Phe	CO	Lys	Val	Hty	MeHty	Ile	[39]
130	Anabaenopeptin 900	900.7	MeHty	CO	Lys	Ile/Leu	Hty	MeAla	MePhe/ Hph	[22]
131	Nodulapeptin 901 / Anabenopeptin 902	902	Phe	CO	Lys	Val	Hph	MeHty	Met	[11, 38]
132	Paltolide C	904.4044	Arg	CO	Lys	Ala	Leu	MeLeu	BrTrp	[15]
133	Anabaenopeptin 905	906.5196	Arg	CO	Lys	Ile	EtHph	MeAsn	Phe	[7]
134	Anabaenopeptin 906	907	Arg	CO	Lys	Ile	MeHty	MeLeu	Phe	[40]
135	Anabaenopeptin KB906	907.5417	Arg	CO	Lys	Ile	Hph	MeHty	Ile	[41]

136	Anabaenopeptin G (1)	909	Arg	CO	Lys	Ile	Hty	MeLeu	Tyr	[42]
137	Anabaenopeptin 908	909.5198	Arg	CO	Lys	Val	Hty	MeHty	Ile	[43]
138	Nodulapeptin B	914.4438	Ile	CO	Lys	Met(O)	Hph	MeHty	AcSer	[44]
139	Anabaenopeptin MM913	914.5031	Tyr	CO	Lys	Ile	Hph	MeHty	Ile	[23]
140	Nodulapeptin 915 A	916	Ile	CO	Lys	MetO	Hph	MeHty	Met	[10]
141	Nodulapeptin 915 B / Anabenopeptin 916	916	Phe	CO	Lys	Val	Hty	MeHty	AcSer	[10, 11]
142	Anabaenopeptin 915	916.4828	Tyr	CO	Lys	Val	Hty	MeHty	Ile	[43]
143	Nodulapeptin 917 / Anabaenopeptin 918	918	Phe	CO	Lys	Val	Hph	MeHty	MetO	[11, 38]
144	Anabaenopeptin KB899	922.4693 $[M+Na]^+$	Tyr	CO	Lys	Val	Hph	MeHty	Ile	[41]
145	Anabaenopeptin H	923.5352	Arg	CO	Lys	Ile	Hty	MeHty	Ile	[45]
146	Keramamide L	927.4541	Phe	CO	Lys	Leu	Leu	MeCTrp	Phe	[46]
147	Oscillamide H	930	Tyr	CO	Lys	MeIle	Ile	MeHty	Ile	[16]
148	Nodulapeptin A	930.4297	Ile	CO	Lys	Met(O2)	Hph	MeHty	AcSer	[44]
149	Anabaenopeptin G (2)	930.4974	Tyr	CO	Lys	Ile	Hty	MeHty	Ile	[45]
150	Nodulapeptin 931	932	Ile	CO	Lys	MetO	Hph	MeHty	MetO	[10]
151	Nodulapeptin 933	934	Phe	CO	Lys	Val	Hty	MeHty	MetO	[10]
152	(-)-Psymbamide A	937.4181	Ile	CO	Lys	Leu	Leu	Me-5′-BrTrp	Phe	[47]
153	Keramamide A	943.4485	Phe	CO	Lys	Leu	Leu	MeCht	Phe	[48]
154	Oscillamide C	957.5198	Arg	CO	Lys	Ile	Hty	MeHty	Phe	[35]
155	Pompanopeptin B	958.529	Ile	CO	Lys	Val	Hty	Me Ahpha	Hty	[49]

Ala = alanine, AcSer = acetyl-serine, Arg = arginine, Asn = asparagine, Bh-Trp = 2-bromo-5-hydroxy-tryptophan, Br-Trp = bromo-tryptophan, Cl-Htyr = chloro-homotyrosine, EtHph = ethyl-homophenylalanine, Gly = glycine, Harg = homoarginine, Hphe = homophenylalanine, Hphe/MePhe = homophenylalanine/methyl-phenylalanine (isobaric compounds), Htyr = homotyrosine, Leu/Ile = leucine/isoleucine (isobaric compounds), Lys = lysine, Met = methionine, MetO = methionine sulfoxide, Met(O)2 = methionine sulfone (S-dioxide), Me-5′-BrTrp = mehtyl-5′-bromo-tryptophan, Me-5′-hydroxyTrp = mehtyl-5′-hydroxy-tryptophan, MeAhpha = N-methyl -2-amino-6-(hydroxyl phenyl) hexanoic acid, MeAla = methyl-alanine, MeAsn = methyl-asparagine, MeCht = 6-chloro-5-hydroxy-N-methyl-tryptophan, MeCTrp = 6-chloro-N-methyl-tryptophan, Me-formyl kyn = methyl-formyl kynurenine, MeGly = methyl-glycine, MeHph = methyl-homophenylalanine, MeHtyr = methyl-homotyrosine, MeLeu/MeIle = methyl-leucine/methyl-isoleucine (isobaric compounds), MeTrp = methyl-tryptophan, OMeArg = arginine methyl ester, OMeGlu = glutamic acid methyl ester, OHTrp = hydroxyl-tryptophan, Phe = phenylalanine, PNV = 5-Phenylnorvaline, PNL = 6-Phenylnorleucine, Ser = serine, Trp = tryptophan, Tyr = Tyrosine, Val = valine.

Table S2. List of the cyanobacterial strains from Greek freshwaters, examined for their ability to produce APs.

Cyanobacterial strain	Growth medium	Maximum growth (day)	Produced APs
Anabaena oscillaroides TAU-MAC 0199	$BG11_0$*	32	-
Calothrix epiphytica TAU-MAC 0399	$BG11_0$	35	-
Chlorogloeopsis fritschii TAU-MAC 0599	$BG11_0$	30	-
Desmonostoc muscorum TAU-MAC 0699	$BG11_0$	29	-
Jaaginema sp. TAU-MAC 0110	BG11	27	-
Jaaginema sp. TAU-MAC 0210	BG11	27	-
Jaaginema sp. TAU-MAC 2210	BG11	26	-
Limnothrix redekei TAU-MAC 0310	BG11	21	-
Microcystis sp. TAU-MAC 0710	BG11	27	-
Microcystis sp. TAU-MAC 1710	BG11	28	-
Microcystis sp. TAU-MAC 1910	BG11	26	-
Microcystis sp. TAU-MAC 2010	BG11	26	-
Microcystis sp. TAU-MAC 2110	BG11	25	-
Microcystis sp. TAU-MAC 2310	BG11	25	-
Microcystis sp. TAU-MAC 2410	BG11	24	-
Microcystis aeruginosa TAU-MAC 0610	BG11	23	-
Microcystis flos-aquae TAU-MAC 0410	BG11	21	-
Microcystis flos-aquae TAU-MAC 1410	BG11	25	-
Microcystis flos-aquae TAU-MAC 1510	BG11	24	-
Microcystis flos-aquae TAU-MAC 1610	BG11	26	-
Microcystis ichtyoblabe TAU-MAC 0510	BG11	29	Anabaenopeptin A, Oscillamide Y
Microcystis viridis TAU-MAC 1810	BG11	23	-
Nodosilinea sp. TAU-MAC 0104	BG11	29	-
Nostoc sp. TAU MAC 0799	$BG11_0$	28	-
Nostoc sp. TAU-MAC 0899	$BG11_0$	27	-
Nostoc elgonense TAU-MAC 0299	$BG11_0$	24	-
Nostoc oryzae TAU-MAC 2610	$BG11_0$	27	-
Nostoc oryzae TAU-MAC 2710	$BG11_0$	27	-
Synechococcus sp. TAU-MAC 0499	BG11	29	-
Synechococcus cf. *nidulans* TAU-MAC 3010	BG11	28	-

* $BG11_0$ is BG11 without nitrogen.

Lake	Sampling date	*Microcystis* sp.	*Dolichospermum* sp.	AP A	AP B	AP F	Osc y	AP 820	AP 842	AP 870	AP 872	AP 886	AP KB906	AP 837	AP 851	AP 894	Number of APs
Amvrakia	10 Aug 1999																4
Amvrakia	19 Aug 1999																1
Amvrakia	19 Aug 1999																3
Kastoria	5 Oct 1995																6
Kastoria	5 Oct 1995																8
Kastoria	3 July 2000																3
Kastoria	20 Sep 2000																4
Kastoria	18 Sep 2014																4
Kastoria	6 Oct 2015																3
Pamvotida	22 July 1999																6
Pamvotida	18 Aug 1999																3
Pamvotida	5 Aug 2000																1
Pamvotida	17 Aug 2000																3
Pamvotida	18 Aug 2000																6
Pamvotida	18 Aug 2000																6
Kerkini	3 Aug 1999																8
Kerkini	26 Aug 1999																6
Zazari	5 Aug 1999																8
Mikri Prespa	5 Aug 1999																6
Mikri Prespa	4 Nov 2014																6
Vistonida	2 Aug 1999																6
Karla	1 July 2015																0
Marathonas	26 Oct 2010																0
		**% Presence**		**35**	**65**	**87**	**87**	**4**	**26**	**13**	**43**	**57**	**4**	**4**	**4**	**9**	

Figure S1. Anabaenopeptins presence in cyanobacterial blooms of Greek freshwaters and the dominant species.

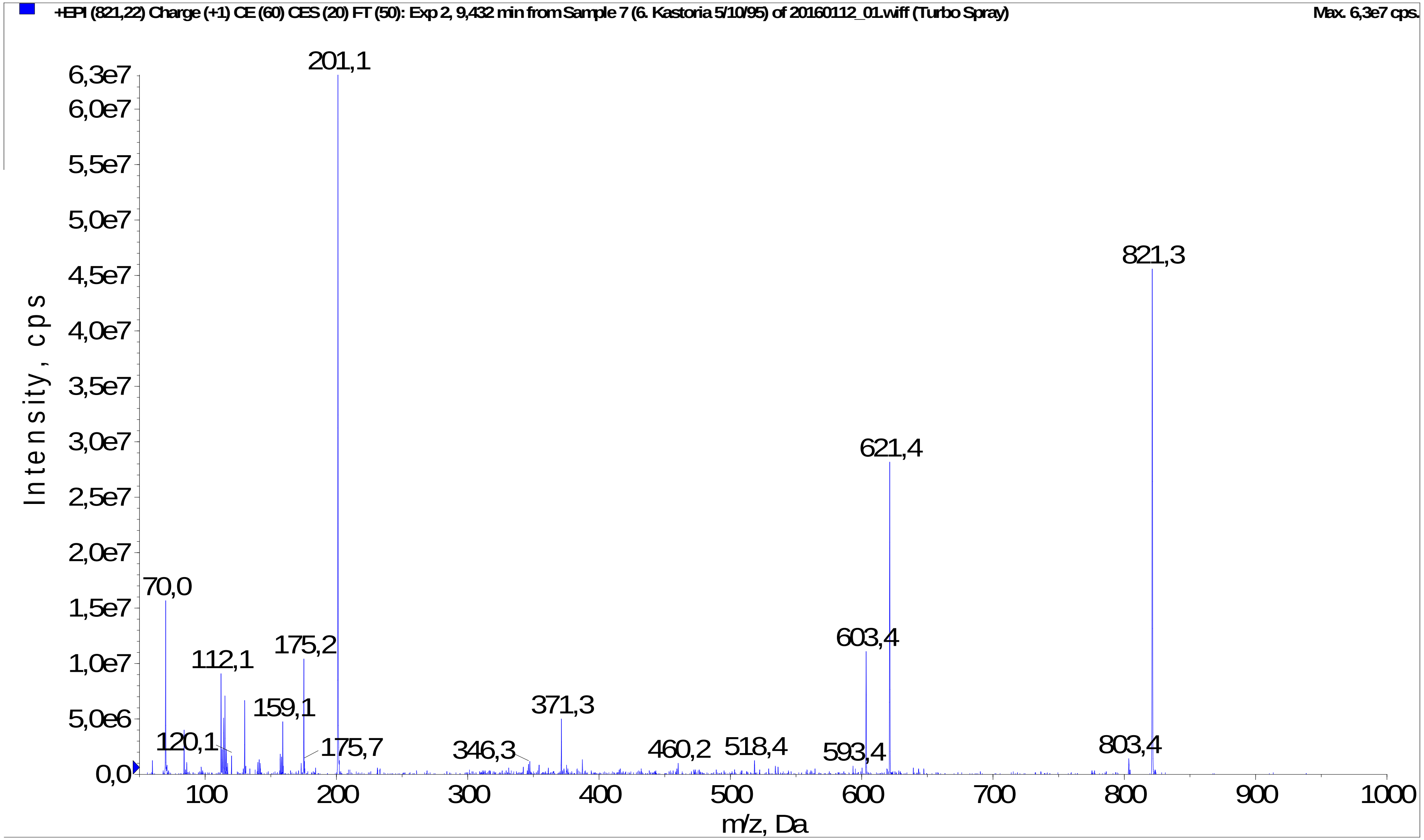

Figure S2. Fragmentation mass spectrum of Anabaenopeptin 820 with precursor ion at *m*/*z* 821 $[M + H]^{+}$.

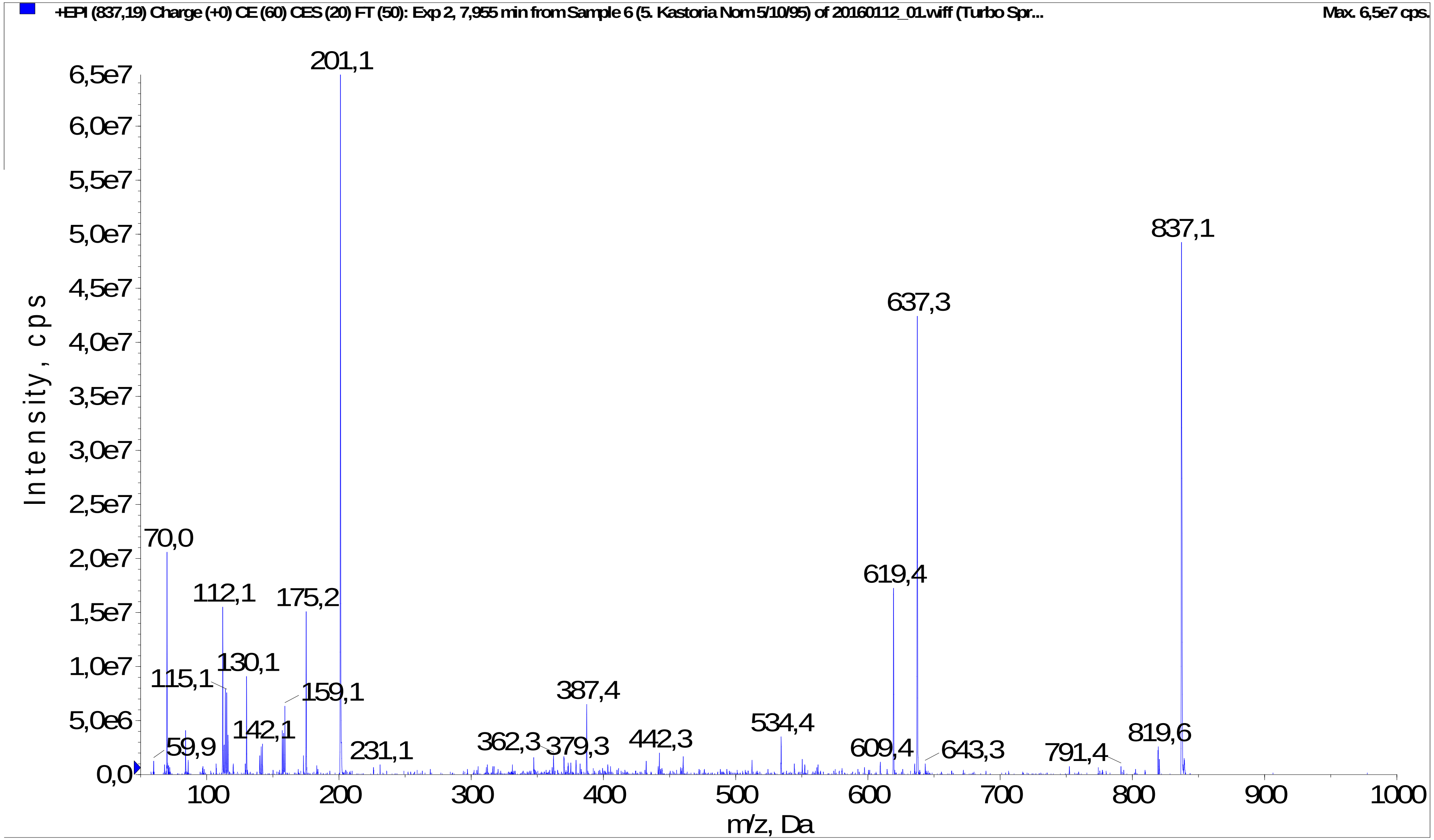

Figure S3. Fragmentation mass spectrum of Anabaenopeptin B with precursor ion at *m*/*z* 837 [M + H]+.

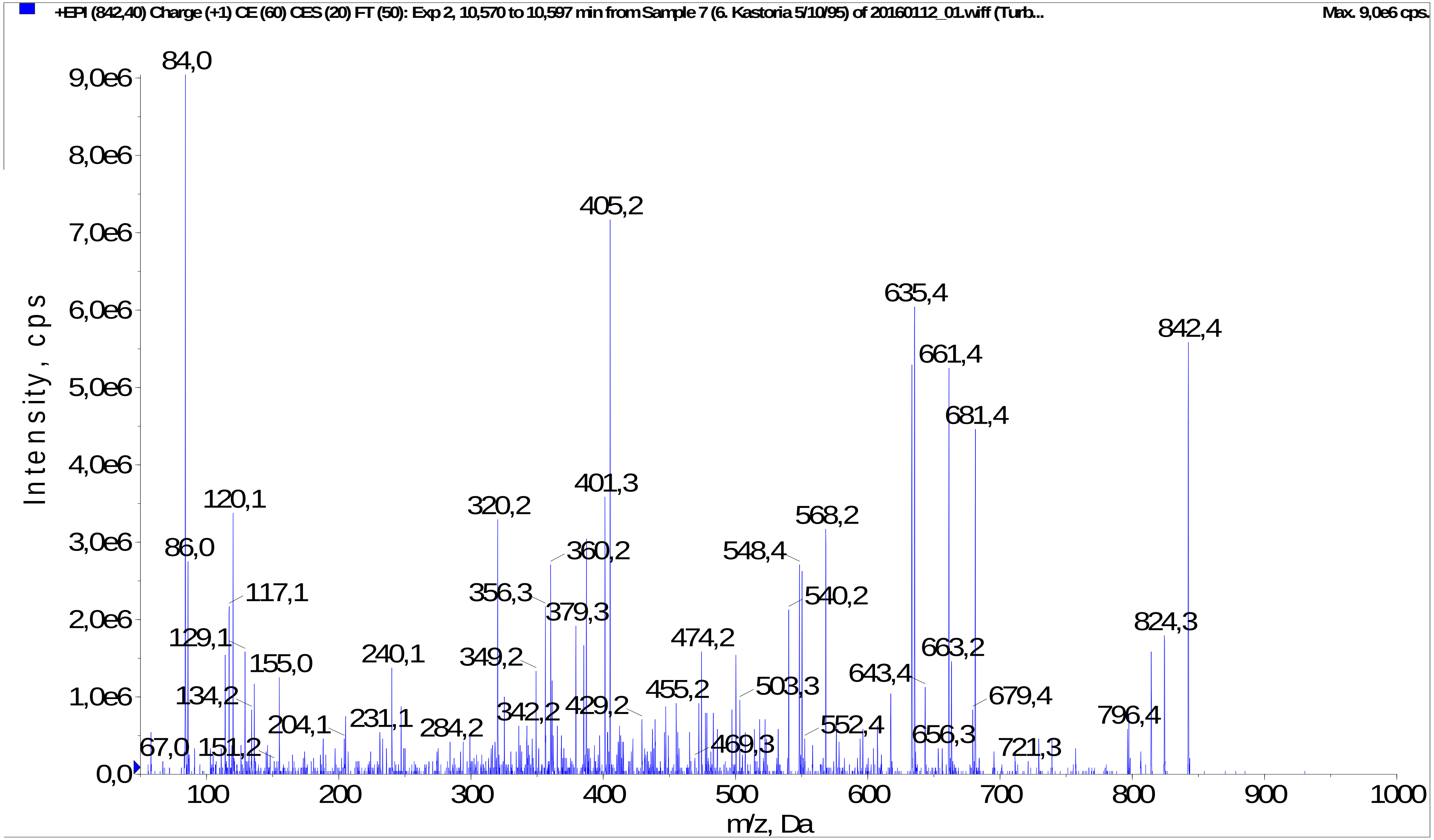

Figure S4. Fragmentation mass spectrum of Anabaenopeptin 842 with precursor ion at *m*/*z* 842 [M + H]+.

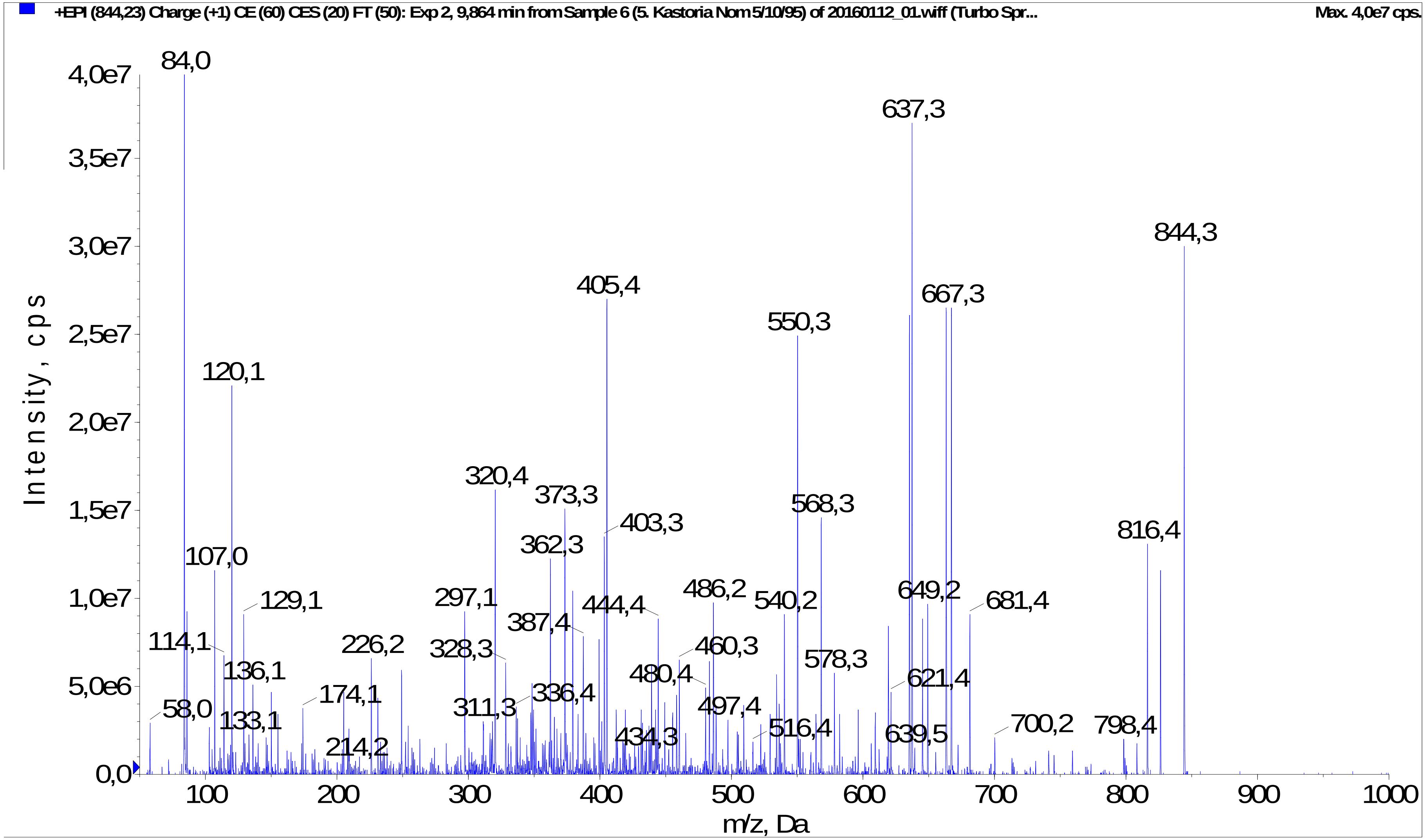

Figure S5. Fragmentation mass spectrum of Anabaenopeptin A with precursor ion at *m*/*z* 844 $[M + H]^{+}$.

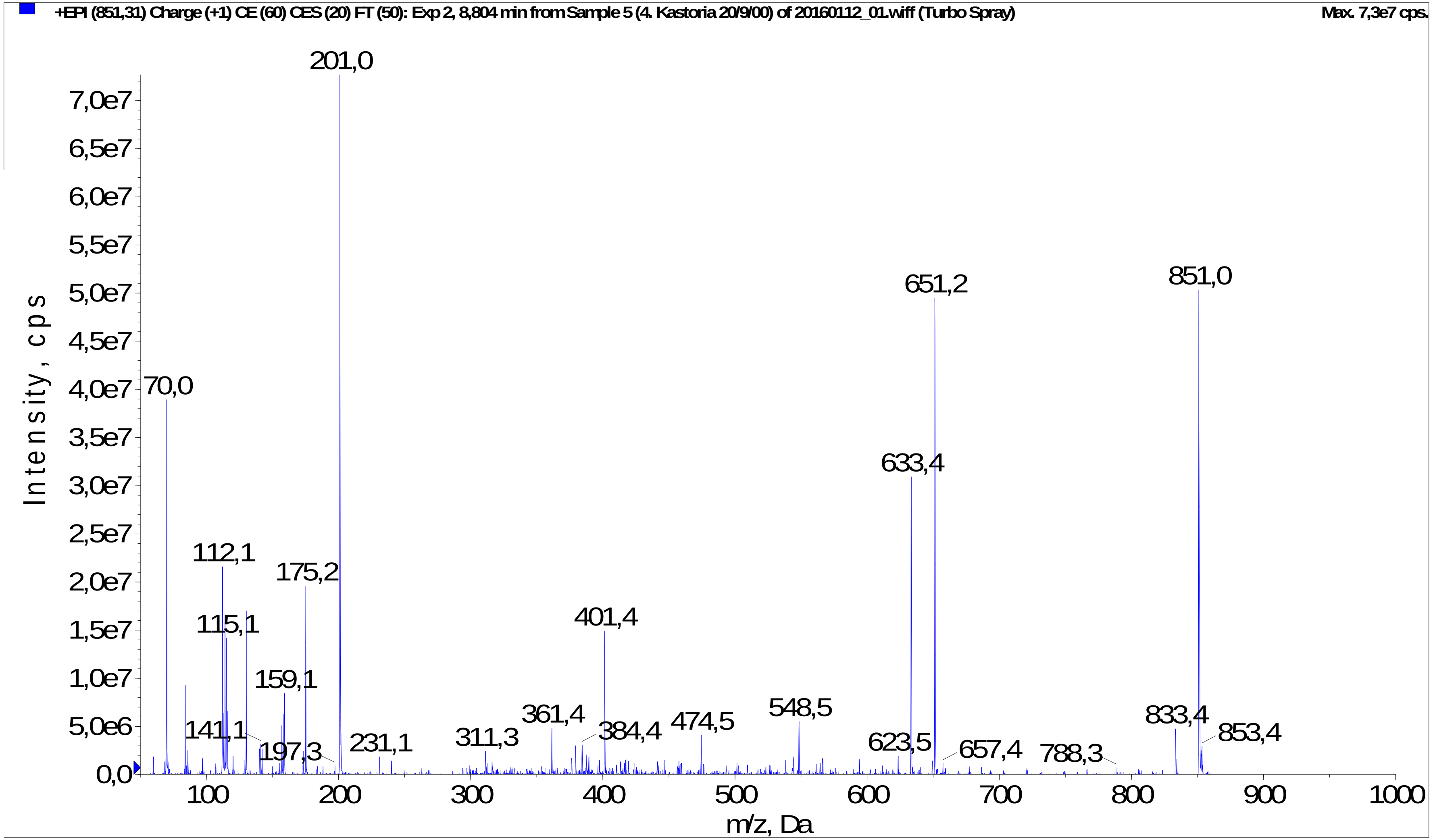

Figure S6. Fragmentation mass spectrum of Anabaenopeptin F with precursor ion at *m/z* 851 $[M + H]^{+}$.

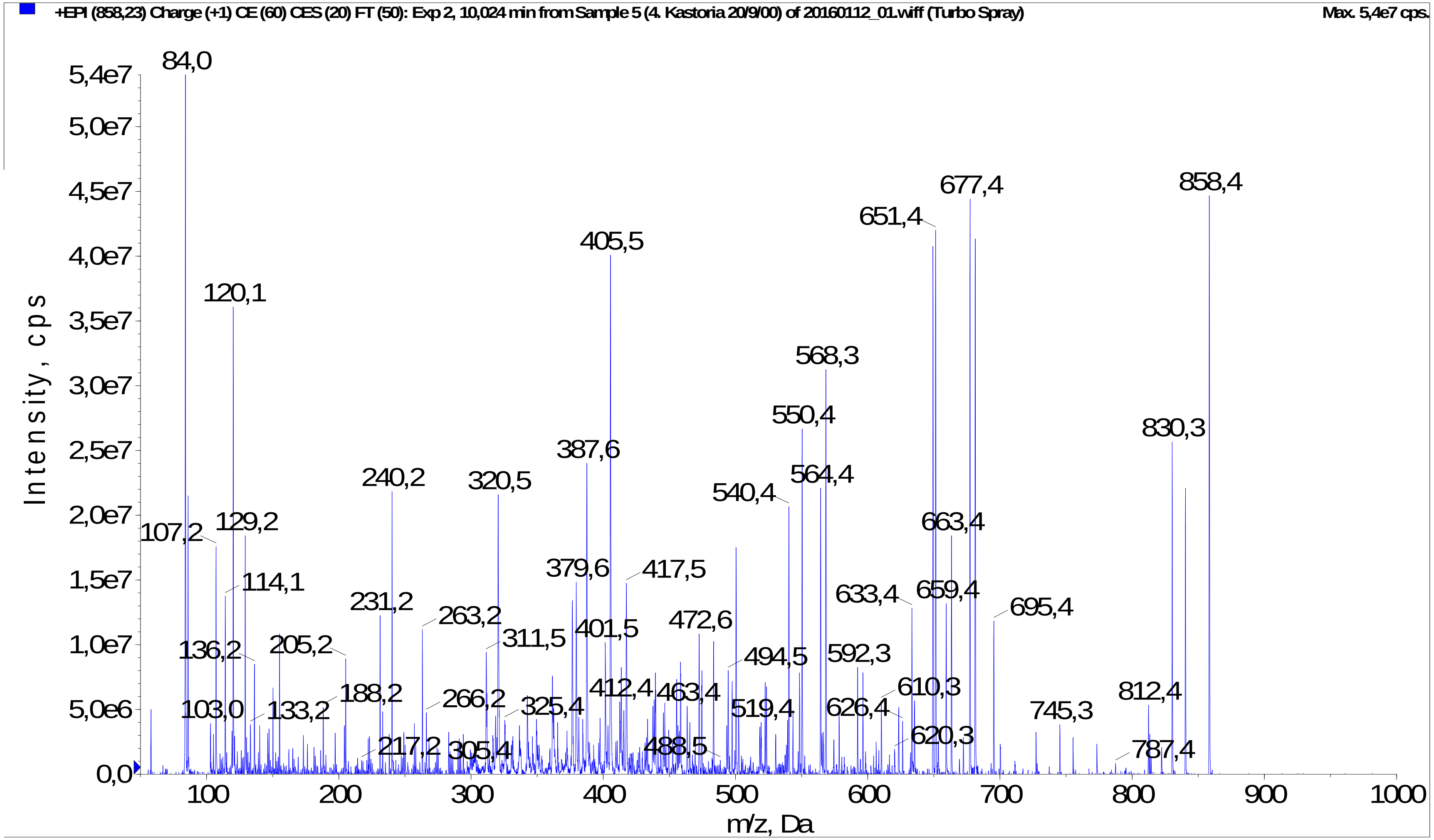

Figure S7. Fragmentation mass spectrum of Oscillamide Y with precursor ion at *m*/*z* 858 $[M + H]^{+}$.

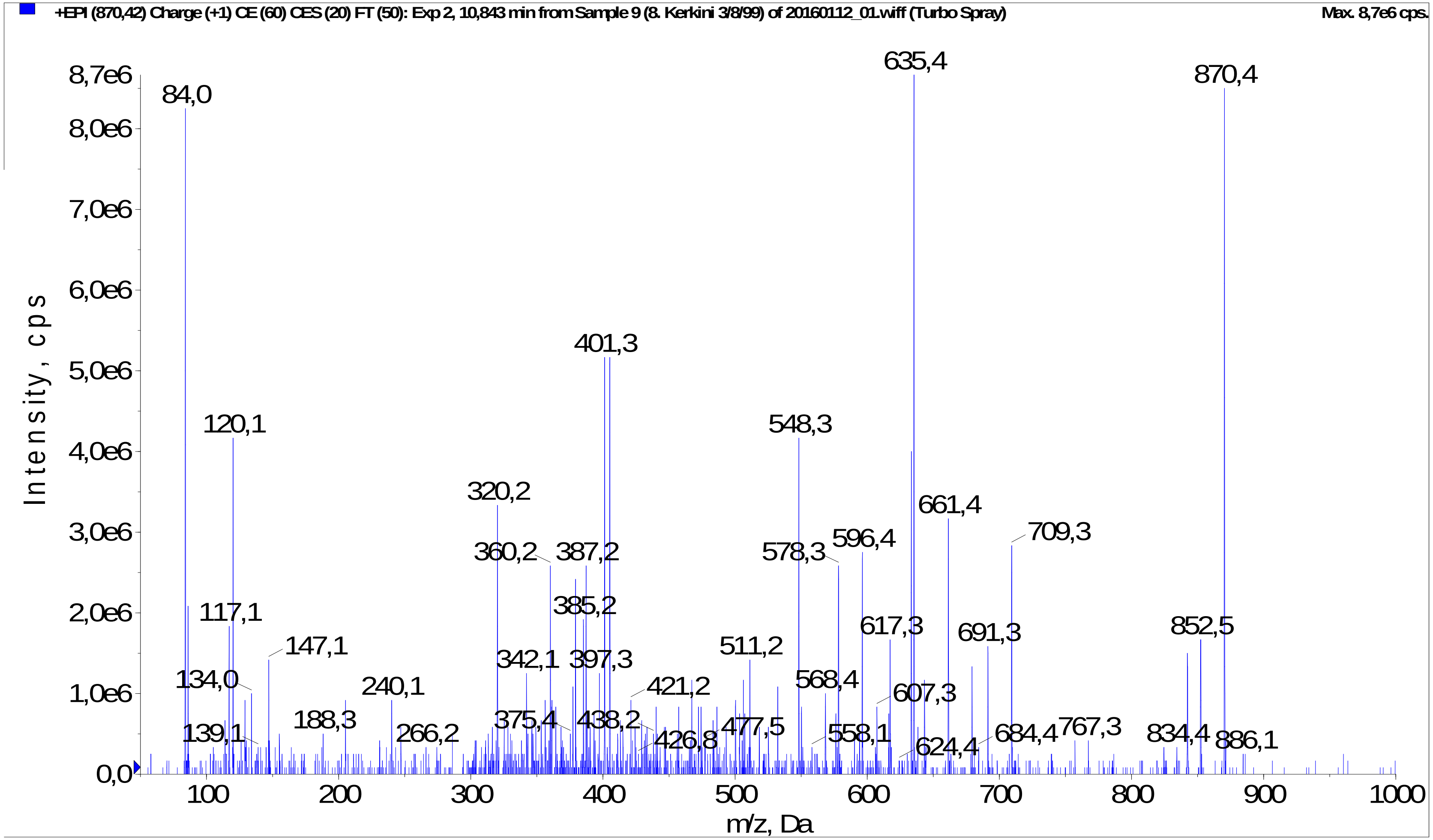

Figure S8. Fragmentation mass spectrum of Anabaenopeptin 870 with precursor ion at *m*/*z* 870 $[M + H]^{+}$.

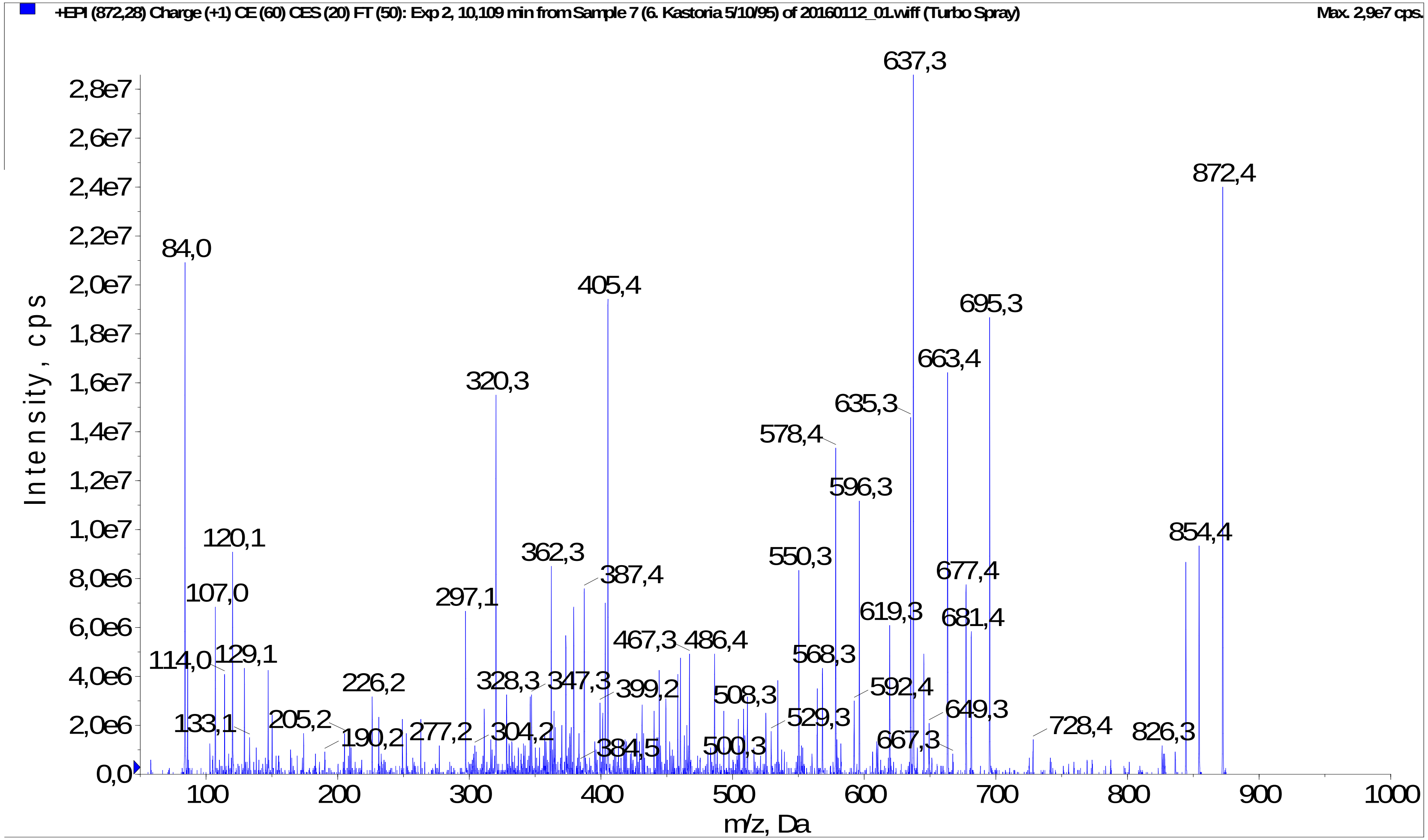

Figure S9. Fragmentation mass spectrum of Anabaenopeptin 872 with precursor ion at *m*/*z* 872 [M + H]+.

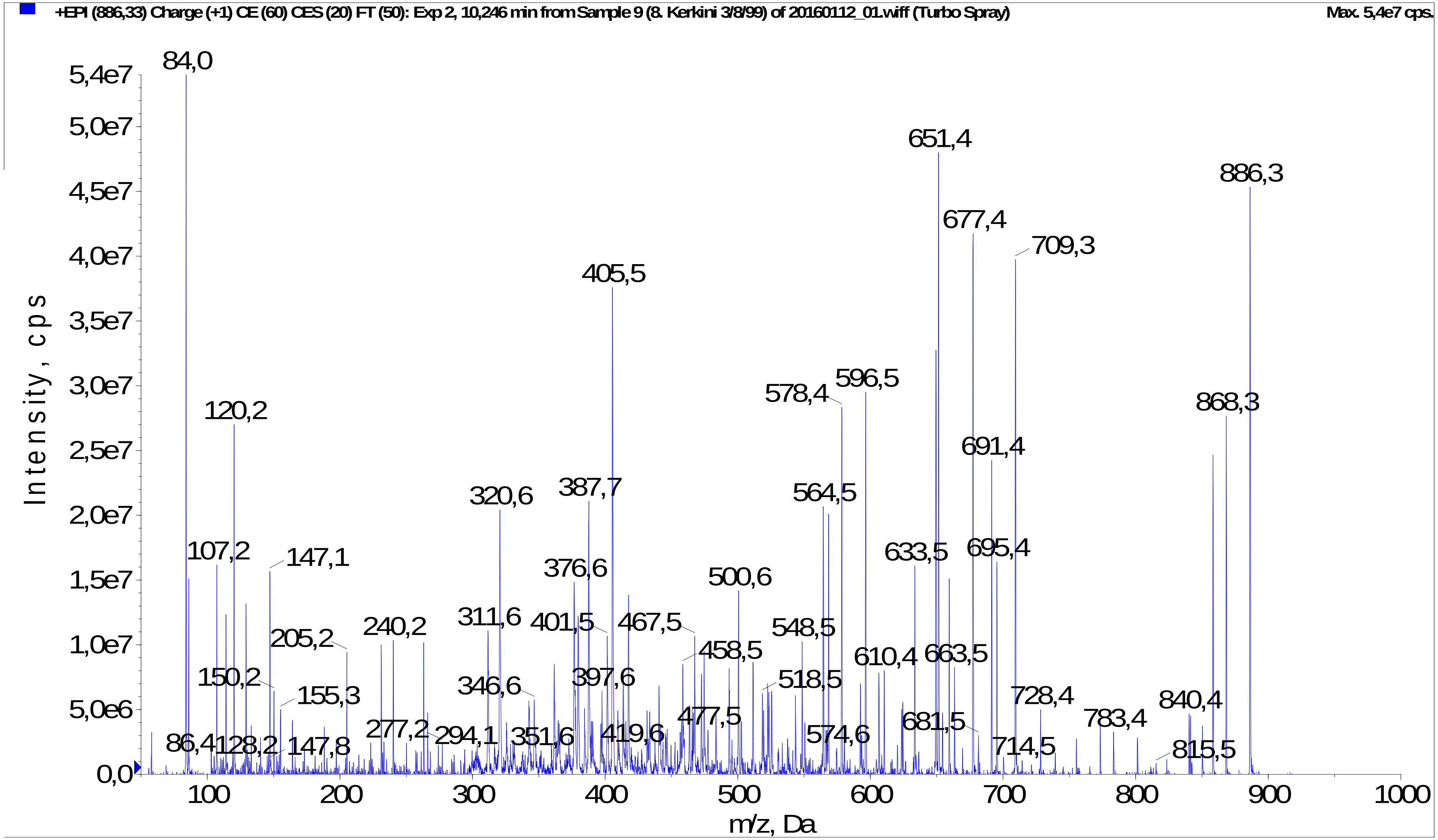

Figure S10. Fragmentation mass spectrum of Anabaenopeptin 886 with precursor ion at *m*/*z* 886 $[M + H]^{+}$.

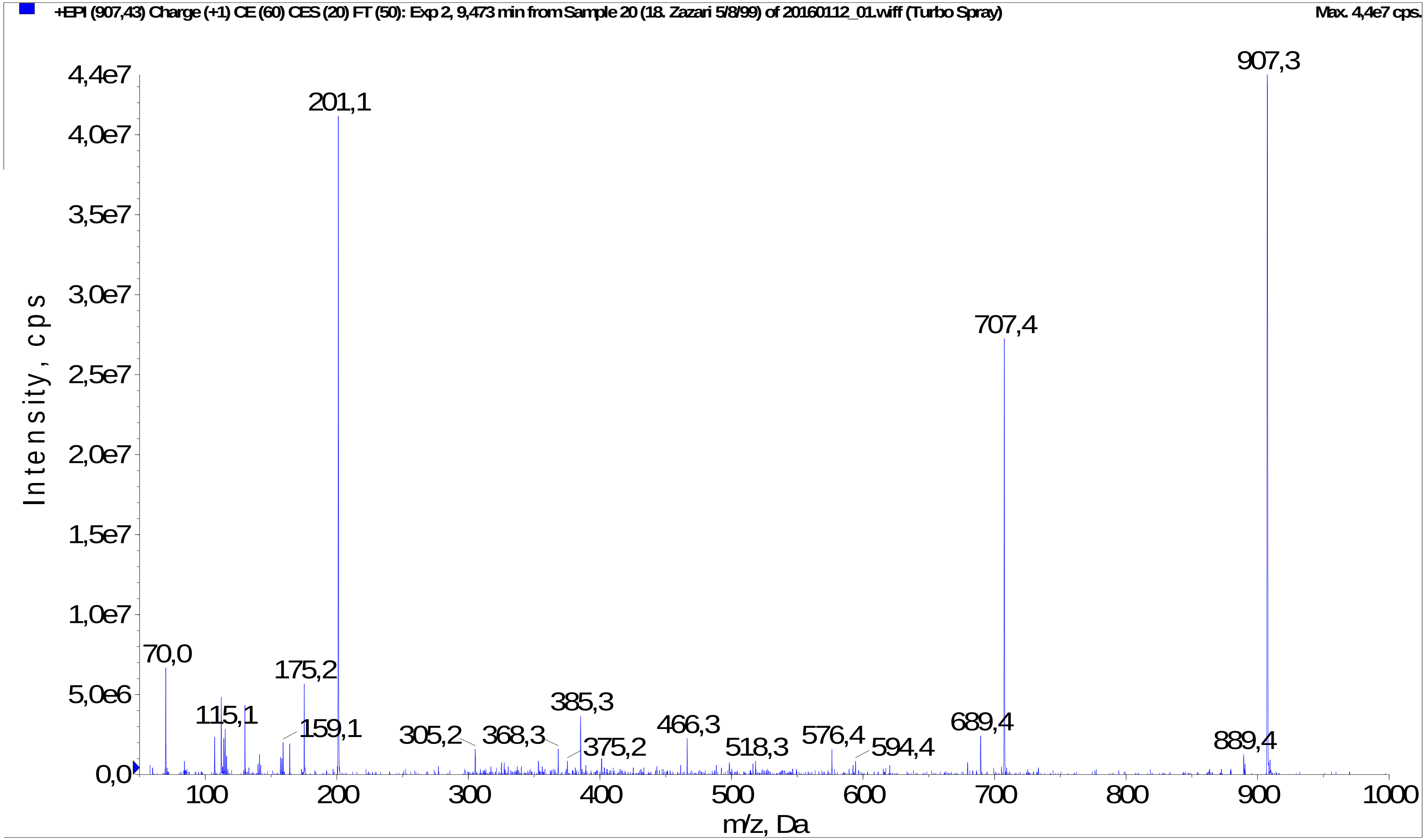

Figure S11. Fragmentation mass spectrum of Anabaenopeptin KB 906 with precursor ion at *m*/*z* 907 $[M + H]^{+}$.

References

1. Harms, H., et al., *Discovery of anabaenopeptin 679 from freshwater algal bloom material: Insights into the structure–activity relationship of anabaenopeptin protease inhibitors.* Bioorganic & Medicinal Chemistry Letters, 2016. **26**(20): p. 4960-4965.
2. Roy-Lachapelle, A., et al., *A Data-Independent Methodology for the Structural Characterization of Microcystins and Anabaenopeptins Leading to the Identification of Four New Congeners.* Toxins, 2019. **11**(11): p. 619.
3. Murakami, M., et al., *New anabaenopeptins, carboxypeptidaze-A inhibitors from the cyanobacterium Aphanizomenon flos-aquae.* Journal of Natural Products, 2000. **63**(9): p. 1280-1282.
4. Shishido, T.K., et al., *Simultaneous Production of Anabaenopeptins and Namalides by the Cyanobacterium Nostoc sp. CENA543.* ACS Chemical Biology, 2017. **12**(11): p. 2746-2755.
5. Saha, S., et al., *Discovery of Unusual Cyanobacterial Tryptophan-Containing Anabaenopeptins by MS/MS-Based Molecular Networking.* Molecules, 2020. **25**(17): p. 3786.
6. Reshef, V. and S. Carmeli, *Schizopeptin 791, a New Anabeanopeptin-like Cyclic Peptide from the Cyanobacterium Schizothrix sp.* Journal of Natural Products, 2002. **65**(8): p. 1187-1189.
7. Sanz, M., et al., *Structural Characterization of New Peptide Variants Produced by Cyanobacteria from the Brazilian Atlantic Coastal Forest Using Liquid Chromatography Coupled to Quadrupole Time-of-Flight Tandem Mass Spectrometry.* Marine Drugs, 2015. **13**(6): p. 3892-3919.
8. Häggqvist, K., et al., *Morphologic, Phylogenetic and Chemical Characterization of a Brackish Colonial Picocyanobacterium (Coelosphaeriaceae) with Bioactive Properties.* Toxins, 2016. **8**(4): p. 108.
9. Mazur-Marzec, H., et al., *Chemical and Genetic Diversity of Nodularia spumigena from the Baltic Sea.* Marine Drugs, 2016. **14**(11): p. 209.
10. Mazur-Marzec, H., et al., *Diversity of Peptides Produced by Nodularia spumigena from Various Geographical Regions.* Marine Drugs, 2013. **11**(1): p. 1-19.
11. Mazur-Marzec, H., et al., *Nodularia spumigena Peptides—Accumulation and Effect on Aquatic Invertebrates.* Toxins, 2015. **7**(11): p. 4404-4420.
12. Fujii, K., et al., *Structural elucidation of cyanobacterial peptides encoded by peptide synthetase gene in Anabaena species.* Tetrahedron, 2002. **58**(34): p. 6863-6871.
13. Fujii, K., et al., *Occurrence of novel cyclicpeptides together with microcystins from toxic cyanobacteria, Anabaena species*, in *Harmful and Toxic Algal Blooms*, T. Yasumoto, Y. Oshima, and Y. Fukuyo, Editors. 1996, Intergovernmental OceanographicCommission of UNESCO: Paris, France. p. 559-562.
14. Guljamow, A., et al., *High-Density Cultivation of Terrestrial Nostoc Strains Leads to Reprogramming of Secondary Metabolome.* Applied and Environmental Microbiology, 2017. **83**(23): p. e01510-17.
15. Plaza, A., et al., *Paltolides A-C, anabaenopeptin-type peptides from the Palau sponge theonella swinhoei.* Journal of Natural Products, 2010. **73**(3): p. 485-488.
16. Spoof, L., et al., *Structures and Activity of New Anabaenopeptins Produced by Baltic Sea Cyanobacteria.* Marine Drugs, 2016. **14**(1): p. 8.
17. Welker, M., et al., *Detection and identification of oligopeptides in Microcystis (cyanobacteria) colonies: Toward an understanding of metabolic diversity.* Peptides, 2006. **27**(9): p. 2090-2103.
18. Schreuder, H., et al., *Isolation, Co-Crystallization and Structure-Based Characterization of Anabaenopeptins as Highly Potent Inhibitors of Activated Thrombin Activatable Fibrinolysis Inhibitor (TAFIa).* Scientific Reports, 2016. **6**.
19. Riba, M., et al., *Chemotyping of terrestrial Nostoc-like isolates from alkali grassland areas by non-targeted peptide analysis.* Algal Research, 2020. **46**: p. 101798.
20. Harada, K.-i., et al., *Two cyclic peptides, anabaenopeptins, a third group of bioactive compounds from the cyanobacteriumAnabaena flos-aquae NRC 525-17.* Tetrahedron Letters, 1995. **36**(9): p. 1511-1514.
21. Rouhiainen, L., et al., *Two Alternative Starter Modules for the Non-Ribosomal Biosynthesis of Specific Anabaenopeptin Variants in Anabaena (Cyanobacteria).* Chemistry and Biology, 2010. **17**(3): p. 265-273.
22. Riba, M., et al., *Microcystis chemotype diversity in the alimentary tract of bigheaded carp.* Toxins, 2019. **11**(5).
23. Zafrir-Ilan, E. and S. Carmeli, *Eight novel serine proteases inhibitors from a water bloom of the cyanobacterium Microcystis sp.* Tetrahedron, 2010. **66**(47): p. 9194-9202.
24. Shin, H.J., et al., *Anabaenopeptins E and F, two new cyclic peptides from the cyanobacterium Oscillatoria agardhii (NIES-204).* Journal of Natural Products, 1997. **60**(2): p. 139-141.
25. Adiv, S. and S. Carmeli, *Protease Inhibitors from Microcystis aeruginosa Bloom Material Collected from the Dalton Reservoir, Israel.* Journal of Natural Products, 2013. **76**(12): p. 2307-2315.
26. Ferranti, P., et al., *A peptidomic approach for monitoring and characterising peptide cyanotoxins produced in Italian lakes by matrix-assisted laser desorption/ionisation and quadrupole time-of-flight mass spectrometry.* Rapid Communications in Mass Spectrometry, 2011. **25**(9): p. 1173-1183.
27. Sano, T. and K. Kaya, *Oscillamide Y, a chymotrypsin inhibitor from toxic Oscillatoria agardhii.* Tetrahedron Letters, 1995. **36**(33): p. 5933-5936.

28. Müller, D., et al., *Brunsvicamides A−C: Sponge-Related Cyanobacterial Peptides with Mycobacterium tuberculosis Protein Tyrosine Phosphatase Inhibitory Activity.* Journal of Medicinal Chemistry, 2006. **49**(16): p. 4871-4878.
29. Schmidt, E.W., M.K. Harper, and D.J. Faulkner, *Mozamides A and B, Cyclic Peptides from a Theonellid Sponge from Mozambique.* Journal of Natural Products, 1997. **60**(8): p. 779-782.
30. Grach-Pogrebinsky, O. and S. Carmeli, *Three novel anabaenopeptins from the cyanobacterium Anabaena sp.* Tetrahedron, 2008. **64**(44): p. 10233-10238.
31. Zi, J., et al., *Lyngbyaureidamides A and B, two anabaenopeptins from the cultured freshwater cyanobacterium Lyngbya sp. (SAG 36.91).* Phytochemistry, 2012. **74**: p. 173-177.
32. Beresovsky, D., et al., *Toxins and biologically active secondary metabolites of Microcystis sp. isolated from Lake Kinneret.* Israel Journal of Chemistry, 2006. **46**(1): p. 79-87.
33. Kodani, S., et al., *Five new cyanobacterial peptides from water bloom materials of lake Teganuma (Japan).* FEMS Microbiology Letters, 1999. **178**(2): p. 343-348.
34. Williams, D.E., et al., *Ferintoic Acids A and B, New Cyclic Hexapeptides from the Freshwater Cyanobacterium Microcystis aeruginosa.* Journal of Natural Products, 1996. **59**(6): p. 570-575.
35. Sano, T., et al., *Isolation of new protein phosphatase inhibitors from two cyanobacteria species, Planktothrix spp.* Journal of Natural Products, 2001. **64**(8): p. 1052-1055.
36. Kobayashi, J., et al., *Konbamide, a novel peptide with calmodulin antagonistic activity from the Okinawan marine sponge Theonella sp.* Journal of the Chemical Society, Chemical Communications, 1991(15): p. 1050-1052.
37. Gesner-Apter, S. and S. Carmeli, *Protease inhibitors from a water bloom of the cyanobacterium Microcystis aeruginosa.* Journal of Natural Products, 2009. **72**(8): p. 1429-1436.
38. Schumacher, M., et al., *New nodulopeptins from Nodularia spumigena KAC 66.* Tetrahedron, 2012. **68**(5): p. 1622-1628.
39. Bober, B., E. Chrapusta-Srebrny, and J. Bialczyk, *Novel cyanobacterial metabolites, cyanopeptolin 1081 and anabaenopeptin 899, isolated from an enrichment culture dominated by Woronichinia naegeliana (Unger) Elenkin.* European Journal of Phycology, 2020: p. 1-11.
40. Puddick, J. and M.R. Prinsep, *MALDI-TOF mass spectrometry of Cyanobacteria: a global approach to the discovery of novel secondary metabolites.* 2008.
41. Elkobi-Peer, S. and S. Carmeli, *New Prenylated Aeruginosin, Microphycin, Anabaenopeptin and Micropeptin Analogues from a Microcystis Bloom Material Collected in Kibbutz Kfar Blum, Israel.* Marine Drugs, 2015. **13**(4): p. 2347-2375.
42. Erhard, M., H. Von Döhren, and P.R. Jungblut, *Rapid identification of the new anabaenopeptin G from Planktothrix agardhii HUB 011 using matrix-assisted laser desorption/ionization time-of-flight mass spectrometry.* Rapid Communications in Mass Spectrometry, 1999. **13**(5): p. 337-343.
43. Okumura, H.S., et al., *Homotyrosine-containing cyanopeptolins 880 and 960 and anabaenopeptins 908 and 915 from Planktothrix agardhii CYA 126/8.* Journal of Natural Products, 2009. **72**(1): p. 172-176.
44. Fujii, K., et al., *Comparative study of toxic and non-toxic cyanobacterial products: Novel peptides from toxic Nodularia spumigena AV1.* Tetrahedron Letters, 1997. **38**(31): p. 5525-5528.
45. Itou, Y., et al., *Anabaenopeptins G and H, Potent Carboxypeptidase A inhibitors from the cyanobacterium Oscillatoria agardhii (NIES-595).* Bioorganic & Medicinal Chemistry Letters, 1999. **9**(9): p. 1243-1246.
46. Uemoto, H., et al., *Keramamides K and L, new cyclic peptides containing unusual tryptophan residue from Theonella sponge.* Tetrahedron, 1998. **54**(24): p. 6719-6724.
47. Robinson, S.J., et al., *Probing the Bioactive Constituents from Chemotypes of the Sponge Psammocinia aff. bulbosa.* Journal of Natural Products, 2007. **70**(6): p. 1002-1009.
48. Kobayashi, J.i., et al., *Keramamide A, a novel peptide from the Okinawan marine sponge Theonella sp.* Journal of the Chemical Society, Perkin Transactions 1, 1991(10): p. 2609-2611.
49. Matthew, S., et al., *Pompanopeptins A and B, new cyclic peptides from the marine cyanobacterium Lyngbya confervoides.* Tetrahedron, 2008. **64**(18): p. 4081-4089.
